# Deoxyribonuclease 1-like 3 Inhibits Hepatocellular Carcinoma Progression by Inducing Apoptosis and Reprogramming Glucose Metabolism

**DOI:** 10.7150/ijbs.57919

**Published:** 2022-01-01

**Authors:** Yusha Xiao, Kang Yang, Pengpeng Liu, Dong Ma, Ping Lei, Quanyan Liu

**Affiliations:** 1 Department of Cardiovascular Surgery, Zhongnan Hospital of Wuhan University, Wuhan 430071, P.R. China.; 2 Department of Hepatobiliary Surgery, Zhongnan Hospital of Wuhan University, Wuhan 430071, P.R. China.; 3 Department of Urology, Renmin Hospital of Wuhan University, Wuhan 430060, China.; 4 Department of Geriatrics, Tianjin Medical University General Hospital Hospital, Tianjin, 300052, P.R. China.; 5 Department of Hepatobiliary Surgery, Tianjin Medical University General Hospital , Tianjin, 300052, P.R. China.

**Keywords:** DNASE1L3, hepatocellular carcinoma, apoptosis, reprogramming glucose metabolism, ZNF384.

## Abstract

HCC has remained one of the challenging cancers to treat, owing to the paucity of drugs targeting the critical survival pathways. Considering the cancer cells are deficient in DNase activity, the increase of an autonomous apoptisis endonuclease should be a reasonable choice for cancer treatment. In this study, we investigated whether DNASE1L3, an endonuclease implicated in apoptosis, could inhibit the progress of HCC. We found DNASE1L3 was down-regulated in HCC tissues, whereas its high expression was positively associated with the favorable prognosis of patients with HCC. Besides, serum DNASE1L3 levels were lower in HCC patients than in healthy individuals. Functionally, we found that DNASE1L3 inhibited the proliferation of tumor cells by inducing G0/G1 cell cycle arrest and cell apoptosis *in vitro*. Additionally, DNASE1L3 overexpression suppressed tumor growth *in vivo*. Furthermore, we found that DNASE1L3 overexpression weakened glycolysis in HCC cells and tissues via inactivating the rate-limiting enzymes involved in PTPN2-HK2 and CEBPβ-p53-PFK1 pathways. Finally, we identified the HBx to inhibit DNASE1L3 expression by up-regulating the expression of ZNF384. Collectively, our findings demonstrated that DNASE1L3 could inhibit the HCC progression through inducing cell apoptosis and weakening glycolysis. We believe DNASE1L3 could be considered as a promising prognostic biomarker and therapeutic target for HCC.

## Introduction

Hepatocellular carcinoma (HCC) is the second leading cause of cancer-related deaths globally 1. Patients are often in the middle and advanced stages, when they are first diagnosed with HCC 2. Advanced stages of HCC require systemic therapies, which show very poor therapeutic records. Moreover, patients undergoing systemic therapies, including radiation, immuno-radiotherapy, and chemotherapy suffer from severe side effects, which range from emesis to tissue damage 3. Therefore, a deeper understanding of molecular pathogenesis and characteristics of liver cancer can help to discovery a better treatment for patients with HCC.

Reprogramming of energy metabolism in tumor cells is now recognized as one of the important features of cancer 4. The transformation from oxidative phosphorylation to glycolysis pathway meets the need of rapid proliferation of HCC cells and provides a good microenvironment for tumor progression. Hexokinase (HK) family catalyzes the first critical step in glycolysis, in which glucose is phosphorylated to glucose 6 phosphate (G-6-P). HK2 serves as one of the HK family members has the highest affinity for glucose and is up-regulated in liver cancer, which is associated with poor prognosis 5. Besides, 6-phosphofructokinase1 (PFK1) served as a rate-limiting enzyme catalyzes the second phosphorylation of glycolysis pathway. PFK1 overexpression is often observed in malignant tumors and is predicted to have a poor prognosis 6. A recent study showed PFK1 was upregulated in HCC and was a predictor of survival and recurrence 7. In our research, we found Deoxyribonuclease1-like 3 (DNASE1L3) may involve in regulating the enzyme activity of HK2 and PFK1.

DNASE1L3 serves as the member of DNA-degrading enzyme: DNases. From a biochemical point of view, solid cancer cells have some remarkable features: they are deficient in DNase activity, they show high rates of glycolysis, cell-free DNA (cfDNA) circulates in cancer patients and induces *in vivo* cell transformation and cancer progression 8. DNases regarded as a promising personalized gene-based therapy for cancer has received a great deal of attention, several apoptotic endonucleases have been identified, the inhibition of both alkaline DNase (DNase I) and acid DNase (DNase II) has been reported in non-necrotic cancer cells at the early stages of experimental carcinogenesis 9. And a number of studies have focused on their individual roles during apoptotic chromatin degradation 10. In this study, we screened overlapping differentially expressed genes (DEG) from public datasets, DNASE1L3 was identified to be associated with malignant progression and prognosis of HCC patients. The two recently published studies also suggested that positive DNASE1L3 expression is an independent prognostic factor for better survival in HCC patients 11, 12.

DNASE1L3 is a secreted DNase that digests DNA in the gastrointestinal tract. DNASE1 and DNASE1L3 together are responsible for the DNase activity in serum 13. Furthermore, endogenous DNASE1L3 is localized to the endoplasmic reticulum (ER) and translocated to the nucleus upon apoptosis induction 14. DNASE1L3 endonuclease activity is not only for the degradation of genomic DNA as a terminal apoptotic step but rather is a participating factor in the overall process of cell death. Overexpression of nuclear DNASE1L3 promotes apoptosis in multiple cell lines, especially during apoptosis mediated by acetaminophen and chemotherapeutic agents 15. Overall, DNASE1L3 plays important roles both extracellularly and intracellularly.

In the present study, we demonstrated that DNASE1L3 acted as a tumor suppressor through loss- and gain-of-function assays *in vivo* and *in vitro* combined with bioinformatics analysis. Moreover, we found serum DNASE1L3 activity was negatively associated with the positron emission tomography (PET)/computed tomography (CT) maximum standardized uptake value (SUVmax) in HCC patients, suggesting that DNASE1L3 is a negative regulator of glucose metabolism. We further demonstrated DNASE1L3 could inhibit glycolysis in the liver cancer cells and promote tricarboxylic acid cycle involved in PTPN2-HK2 and CEBPβ-p53- PFK1 pathways. We also discovered ZNF384 was a negative regulatory transcriptional factor upstream of DNASE1L3. HBV X protein (HBx) could inhibit the mRNA level of DNASE1L3 by up-regulating ZNF384, thus promoting the development of HCC.

## Materials and Methods

### Bioinformatics

Public tumor microarray databases of HCC were analyzed as described in the Supplementary Material.

### Cell culture and reagents

Human HCC cell lines, including Huh7, SK-hep1, HepG2, Hep3B, HCCLM3, and immortalized human liver cell line HL-7702 (L02) were cultured as described in the Supplementary Material.

### Patient and clinical sample collection

A total of 152 postoperative HCC patients in two independent cohorts were analyzed as described in the Supplementary Material. Ethical approval was obtained from the Zhongnan Hospital of Wuhan University Research Ethics Committee. Informed consent was procured from each patient.

### Total RNA isolation and quantitative real-time PCR

RNA isolation, complementary DNA synthesis, and quantitative real-time (PCR qRT-PCR) reactions were performed as described in the Supplementary Material. The primers involved in this research are listed in Supplementary Table 1.

### Plasmid construction, lentiviral construction, RNA interference, and cell transfections

DNASE1L3 was inserted into pcDNA3.1 vector purchased from GeneCreate (Wuhan, China) and transfected into Huh7 and HCCLM3 cells using Lipofectamine 3000 reagent (Thermo Fisher Scientific; Waltham, USA). Other details can be found in the Supplementary Material.

### Immunohistochemistry and immunofluorescence

Immunohistochemistry (IHC) and immunofluorescence (IF) were conducted as described in the Supplementary Material. Antibodies involved in the assays are shown in Supplementary Table 2.

### Cell proliferation assay

After cells were transfected for 48 ch with the desired siRNA or plasmid, CCK8 and colony formation assays were described in detail in the supplementary methods.

### Flow cytometry analysis

FACSCalibur flow cytometer (BD Biosciences, USA) was used to analyze the cells. Detailed information is provided in Supplementary Material.

### Cell migration and invasion assay

Cell migration and invasion were measured by scratch assay and transwell assay. The details are provided in Supplementary Material.

### Western blotting

The detailed procedure of western blotting is described in Supplementary Material. Information of antibodies is provided in Supplementary Table 2.

### Label-free quantitative proteomics

Label-free quantitative proteomics was performed by GeneCreate (Wuhan, China). Mass spectrometry data were collected using Thermo Scientific Q Exactive (Waltham, USA) and a liquid-mass system. The specific operation steps are described in detail in the Supplementary Material.

### Metabolite quantification by liquid chromatography-tandem mass spectrometry

Metabolite quantification was conducted by Biotree biotech (Shanghai, China) using liquid chromatography-tandem mass spectrometry (LC-MS/MS). The detailed methods are provided in Supplementary Material.

### Chromatin immunoprecipitation assay

Chromatin immunoprecipitation (ChIP) assay was performed using the Magna ChIP-seq™ Chromatin Immunoprecipitation Kit (Millipore; Billerica, USA) according to manufacturer's instructions. The detailed methods are provided in Supplementary Material.

### Dual-luciferase reporter activity assay

Luciferase activity was measured using a Dual-Luciferase Assay Kit (Promega; Madison, WI, USA) according to the manufacturer's instructions. The detailed methods are provided in Supplementary Material.

### Tumorigenesis assay

Five-week-old male BALB/c nude mice were purchased from the Animal Center of the Chinese Academy of Medical Sciences (Beijing, China). Subcutaneous xenotransplanted tumor and orthotopic HCC mouse models were used to analyze HCC tumor growth. For details, see Supplementary Material.

### Hematoxylin-eosin staining

Hematoxylin-eosin** (**H&E) staining was performed as described in the Supplementary Material.

### DNASE1L3 ELISA assay

DNASE1L3 enzyme-linked immunosorbent assay (ELISA) kit was used to analyze the serum DNASE1L3 levels. Detailed information is available in Supplementary Material.

### Statistics

Statistical analyses were performed using SPSS v. 24.0 (IBM Corp., Armonk, NY, USA). Data are presented as mean ±SD (standard deviation) from at least three independent experiments. The significance of the differences between the groups was determined using Student's *t*-test, whereas linearity was evaluated by Pearson's correlation coefficient. Pearson's χ^2^ test was applied to compare categorical variables, and *p* < 0.05 was considered statistically significant. Survival curves were constructed using the Kaplan-Meier method. Univariate and multivariate analyses were completed using the Cox proportional hazard regression model.

## Results

### DNASE1L3 was significantly down-regulated in HCC with decreased expression predicting poor prognosis

To recognize differentially expressed genes (DEGs) in HCC tissues, we extracted HCC databases of GSE76427, GSE64041, GSE57957, and GSE45436 from the Gene Expression Omnibus (GEO). After cross-examining these datasets, 21 mRNAs were identified as depicted in the Venn diagram (Supplementary Fig. 1A). Heatmap plots (Supplementary Fig. 2A-D) revealed 21 genes with significantly differential expression (fold changes ≥ 2.0 and *p* < 0.05) between groups that were consistently up-regulated or were down-regulated in the four datasets. These different mRNA transcriptions were further confirmed through comparing the expression between tumor and healthy samples in The Cancer Genome Atlas (TCGA) Liver hepatocellular carcinoma (LIHC) dataset (Supplementary Fig. 1B). Among these DEGs, DNASE1L3 was the only DNase member, which was down-regulated in 50 pairs of HCC tissues compared with surrounding healthy liver samples extracted from the TCGA-LIHC dataset (Supplementary Fig. 2E). Furthermore, DNASE1L3 expression was tightly associated with tumor grades of HCC (Supplementary Fig. 2F). Subsequently, its capabilities in prognosticating HCC was assessed to determine disease-free survival (DFS) and overall survival (OS) using Kaplan-Meier plots. We found that reduced expression of DNASE1L3 was significantly associated with poorer survival in HCC patients (Supplementary Fig. 2G and H). We also investigated the role of other apoptotic endonucleases, including DNase1, DNase2, and DFFB, and noticed these genes could not predict the prognosis of HCC (Supplementary Fig. 3). Finally, we explored the effect of DNASE1L3 expression on the prognosis of other malignancies in the TCGA database. The results suggested that high DNASE1L3 expression could significantly improve the OS of patients with lung adenocarcinoma, pancreatic cancer, sarcomaand kidney cancer (Supplementary Fig. 4).

**Figure 1:**
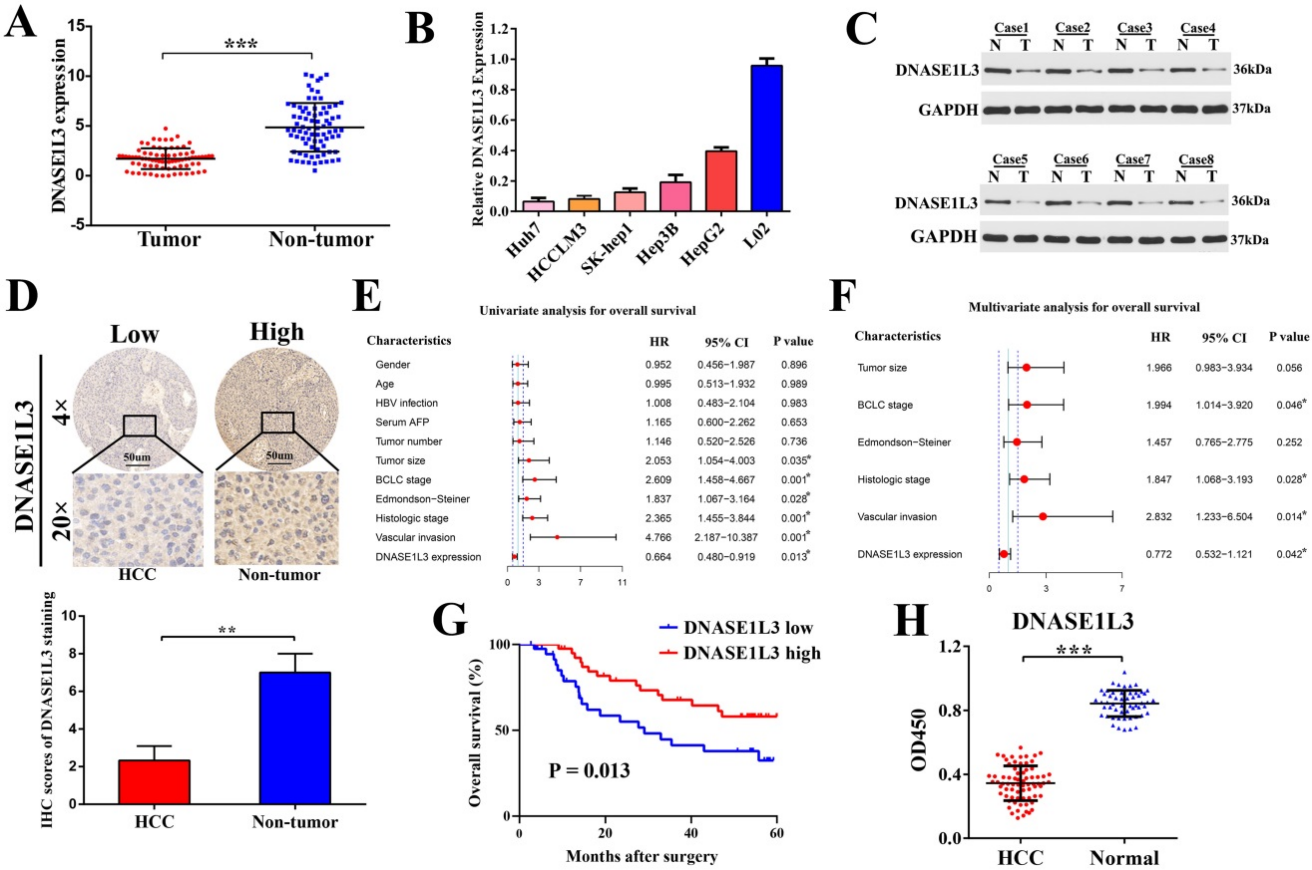
** DNASE1L3 was downregulated in HCC tissues and was linked to favorable prognosis. (A)** DNASE1L3 expression was determined in 80 pairs of HCC samples and corresponding healthy tissues via RT-qPCR **(B)** DNASE1L3 expressions were determined in Huh7, HCCLM3, HepG2, Hep3B, SK-Hep1 and immortalized normal human hepatic cell (L02) lines as well as THP1 via RT-qPCR **(C)** Western blotting showed DNASE1L3 protein levels in 8 pairs of human HCC tumor samples and adjacent healthy tissues. **(D)** Representative immunohistochemistry images for DNASE1L3 expression in 18 pairs of HCC and paired paracancerous liver tissues. **(E-F)** Univariate and multivariate analyses of the association between clinicopathological features and the overall survival rate of HCC patients. **(G)** Kaplan-Meier analysis of OS according to DNASE1L3 expression levels in 80 patients with HCC.** (H)** ELISA of serum DNASE1L3 in 54 normal human and 72 patients with liver cancer. *p < 0.05, **p < 0.01, ***p < 0.001.

To verify the bioinformatics analysis, DNASE1L3 expression and its relationship to clinicopathological characteristics were then evaluated in 80 patients with HCC in an independent cohort (cohort Ⅰ). We found there was a marked downregulation of DNASE1L3 mRNA levels in tumor tissues in contrast to the corresponding adjacent tissues (Fig. 1A). mRNA levels of DNASE1L3 was then quantified across different human liver cell lines. Hepatoma cells lines were found to possess depressed DNASE1L3 expression compared with immortalized liver cell line L02 (Fig. 1B). Western blotting (Fig. 1C) and immunohistochemistry (Fig. 1D) then confirmed that the protein expression of DNASE1L3 in HCC tissues was decreased compared with paired adjacent non-tumor tissues. DNASE1L3 expression was strongly linked to histologic grade (*p*=0.0062), Edmondson-Steiner grade (*p* = 0.0098), BCLC stage (*p* = 0.044), tumor size (*p* = 0.012), and vascular invasion (*p* = 0.034) (Supplementary Table 3). Furthermore, the Cox regression analysis model highlighted DNASE1L3 expression to possess the ability to independently predict poor prognosis in HCC patients through univariate (Fig. 1E) and multivariate (Fig. 1F) analyses. Kaplan-Meier survival curve analyses uncovered that OS was markedly shorted in HCC patients who possessed lower DNASE1L3 levels in contrast to those who had high levels of this protein (*p* = 0.013) (Fig. 1G). Finally, to investigate the potential of DNASE1L3 as a novel biomarker for human HCC, we examined the serum levels of DNASE1L3 in 72 patients with HCC and 54 healthy individuals (cohort Ⅱ) by ELISA. The results demonstrated that serum DNASE1L3 activity was higher in healthy individuals than in patients with HCC (Fig. 1H), implying that DNASE1L3 was a potential serum biomarker for diagnosing HCC.

### DNASE1L3 repressed HCC cell invasion and proliferation, arrested cell cycle, and promoted cell apoptosis *in vitro*

**Figure 2:**
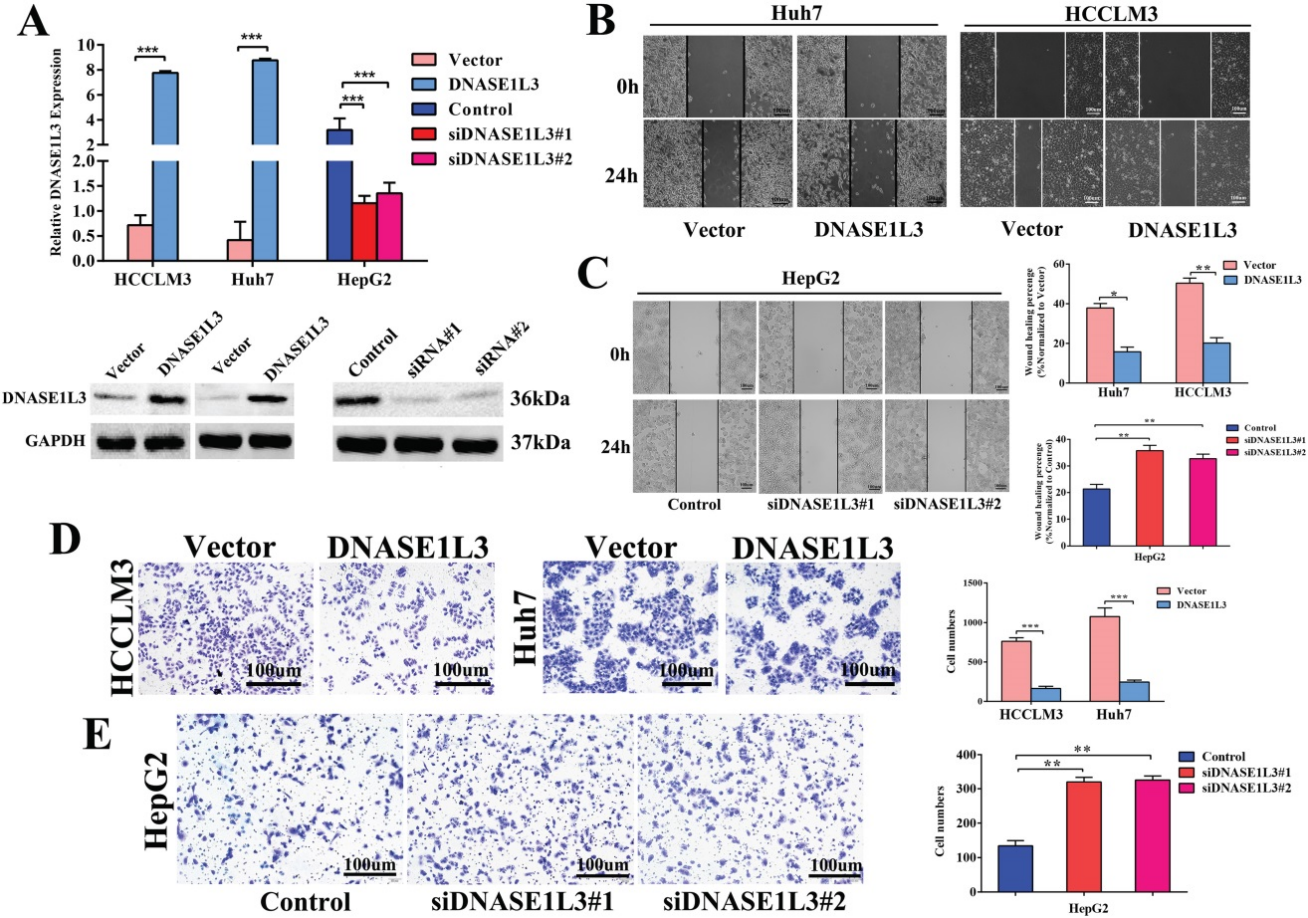
** DNASE1L3 overexpression inhibited cell invasion and cell migration *in vitro*. (A)** DNASE1L3 overexpression and knock-down efficacy were determined in Huh7, HepG2 and HCCLM3 cell lines via western blotting and RT-qPCR. **(B-C)** Wound healing assay was conducted both in DNASE1L3-overexpressing Huh7 and HCCLM3 cell lines and DNASE1L3 knock-down HepG2 cell line. **(D-E)** Transwell assay was performed both in DNASE1L3-overexpressing Huh7 and HCCLM3 cells and DNASE1L3 knock-down HepG2 cells. *p < 0.05, **p < 0.01, ***p < 0.001.

**Figure 3:**
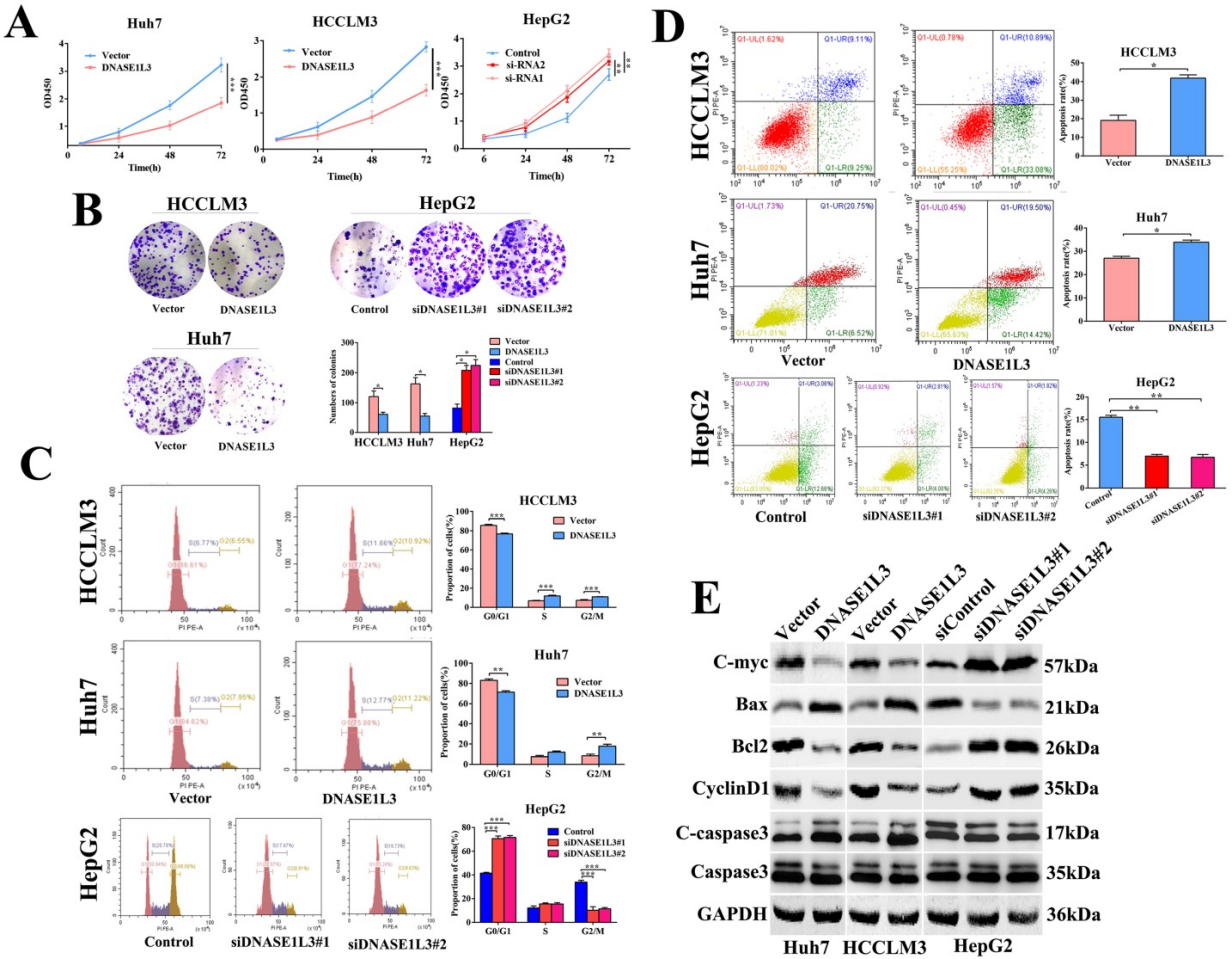
** DNASE1L3 suppressed cell proliferation, halted the cell cycle, and stimulated cell apoptosis. (A-B)** CCK8 assay and colony formation assay were carried out in order to assess the impact of DNASE1L3 expression modification on the proliferative capabilities of DNASE1L3-overexpressing Huh7 and HCCLM3 cells and DNASE1L3 depleted HepG2 cells. **(C)** Flow cytometric analysis assessed the impact of various DNASE1L3 expression levels on the cell cycle in DNASE1L3-overexpressing Huh7 and HCCLM3 cells or DNASE1L3 silenced HepG2 cells.** (D)** Flow cytometric analysis to assess cell apoptosis was performed in DNASE1L3-overexpressing Huh7 and HCCLM3 cells or DNASE1L3 knock-down HepG2 cells.** (E)** Western blotting analysis was performed to determine the impact of various DNASE1L3 levels on C-myc, Bcl2, Cyclin D1, Bax, and cleaved-caspase 3 in DNASE1L3-overexpressing Huh7 and HCCLM3 cells or DNASE1L3 knock-down HepG2 cells. *p < 0.05, **p < 0.01, ***p < 0.001.

To explore the functional effects of DNASE1L3 in different HCC subtypes, we chose Huh7 and HCCLM3 cells as DNASE1L3 overexpression models, while HepG2 cell was chosen as the knock-down model based on DNASE1L3 expression level in 5 HCC cell lines for subsequent experiments. After the plasmid, control plasmid, and two siRNAs (Designated no. 1 and no. 2) were transfected into the cells, respectively, the overexpression and knock-down efficacy were estimated by RT-qPCR and western blotting (Fig. 2A). Next, the cell migratory and invasive abilities were examined by the wound healing assay (Fig. 2B and C) and transwell assay (Fig. 2D and E), respectively. The cell proliferative ability was assessed using the CCK8 assay (Fig. 3A) and colony formation assay (Fig. 3B), separately. The results indicated that the overexpression of DNASE1L3 markedly reduce the abilities of Huh7 and HCCLM3 cells to invade and migrate, whereas its reduced expression promoted HepG2 cells migration and invasion. CCK8 and colony formation assays showed that up-regulation of DNASE1L3 could significantly reduce the growth rate and cell proliferation in Huh7 and HCCLM3 cell lines, whereas the results were reversed in HepG2 cell line with knock-down of DNASE1L3 (Fig. 3A and B).

The impact of DNASE1L3 on cell cycle and apoptosis was assessed by flow cytometry. The DNASE1L3 overexpression raised G2/M phase cell proportion (Fig. 3C) and induced apoptosis of Huh7 and HCCLM3 cells (Fig. 3D). As expected, its down-regulation diminished the proportion of cells in the G2/M phase (Fig.3C) and suppressed the apoptosis of HepG2 cells (Fig. 3D). Western blotting was used to detect changes in protein expression levels related to proliferation, cell cycle, and apoptosis in three HCC cell lines and demonstrated that augmented DNASE1L3 expression dramatically suppressed the protein levels of c-myc, Bcl2, and Cyclin D1, whereas protein levels of Bax and cleaved-caspase 3 were raised (Fig. 3E). These results indicated that DNASE1L3 inhibited tumor cell proliferation, migration, and invasive capabilities, which may be attributed to G0/G1 cell cycle arrest and cell apoptosis.

### DNASE1L3 inhibited tumor growth *in vivo*

**Figure 4:**
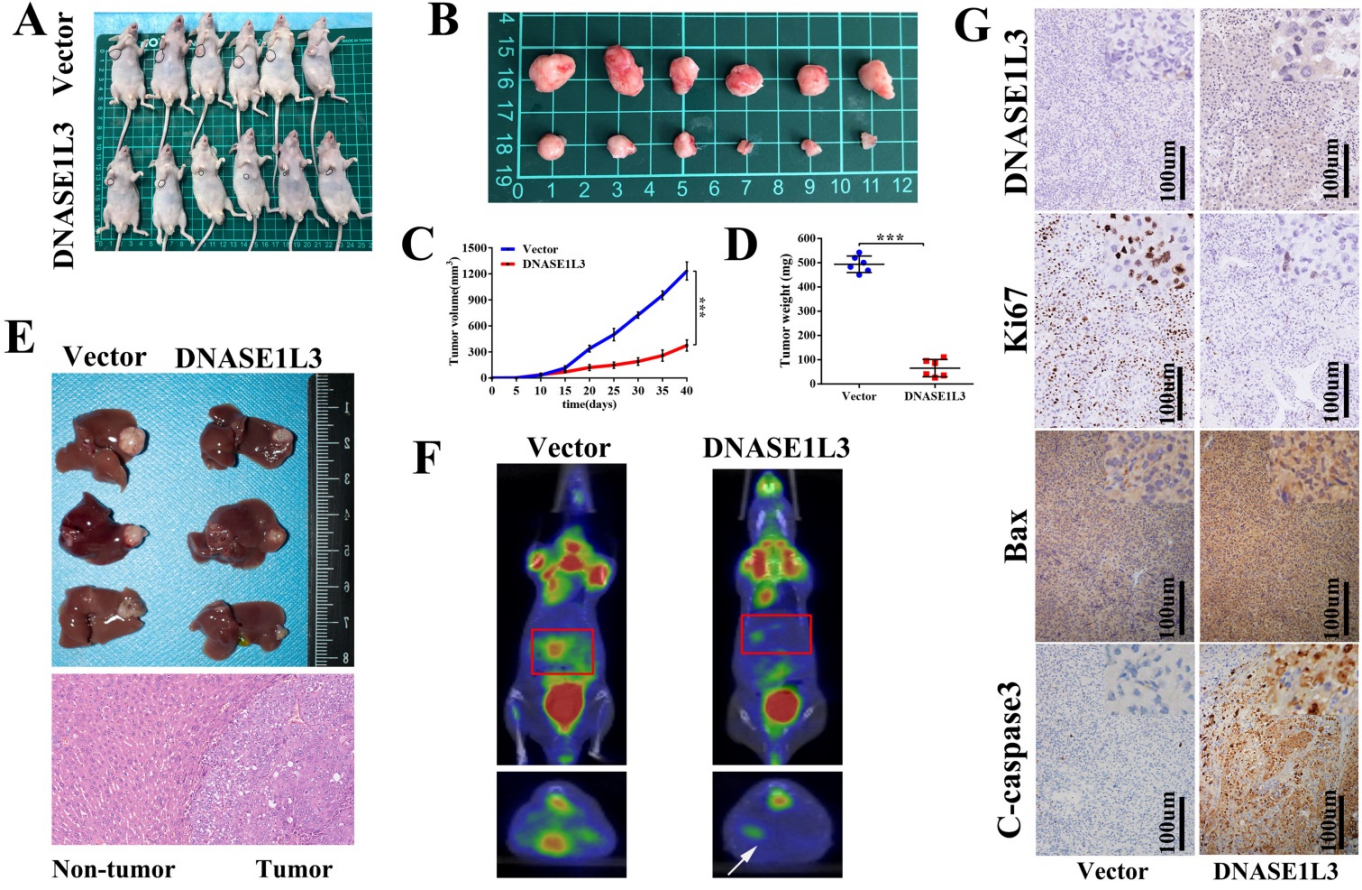
** DNASE1L3 overexpression inhibited HCC tumor growth and induced apoptosis *in vivo*. (A-B)** Images of xenograft tumors in nude mice from the treatment group (n = 6) and control group (n = 6) after subcutaneous injection of HCCLM3 cells transfected with stably overexpressing DNASEL13 plasmids on day 42.** (C)** Growth curves of tumor of nude mice subcutaneously injected with HCCLM3 cells stably overexpressing DNASE1L3 or control cells were calculated. **(D)** Final tumor weights **(E)** Representative images of an orthotopic transplantation tumor model of HCC in nude mice and H&E staining of the resultant liver tumor. **(F)** Characteristic images of an orthotopic transplantation tumor model of HCC in PET/CT.** (G)** Immunohistochemistry was investigate the impact of DNASE1L3 overexpression on the protein levels of Ki67, cleaved caspase 3, Bax, and DNASE1L3 *in vivo*. *p < 0.05, **p < 0.01, ***p < 0.001.

To validate the *in vitro* phenotype of DNASE1L3, tumor growth suppression was studied *in vivo*. HCCLM3 cells stably overexpressing DNASE1L3 or control HCCLM3 cells were constructed and subcutaneously inoculated into the armpit of male nude mice to generate a HCC xenograft (Fig. 4A and B). The overexpression of DNASE1L3 intensely suppressed tumor growth (Fig. 4C) and decreased tumor weight (Fig. 4D) of the HCC xenograft. To further confirm this finding, HCCLM3 cells subcutaneous xenografts were isolated and implanted into the livers of nude mice to establish a HCC orthotopic xenograft model. After six weeks, PET/CT operated by the Union Hospital PET Center of Tongji Medical College (Wuhan, China) was used to evaluate the size of the liver lesions. Nude mice who possessed stably-overexpressed DNASE1L3 cells demonstrated stunted liver tumor growth and lower glucose metabolic activity of the liver lesions (Fig. 4F). Harvested liver tumor sample analyses found overexpression of DNASE1L3 inhibited liver tumor growth in nude mice (Fig. 4E). Following IHC analysis using antibodies for the specific detection of DNASE1L3, Bax and C-Caspase3 were found to be markedly augmented while Ki67 was suppressed (Fig. 4G). These results suggested that DNASE1L3 could inhibit tumor growth *in vivo.*

### Inhibitory effect of DNASE1L3 on tumor cells was closely related to JAK/STAT and glycolysis pathways

**Figure 5:**
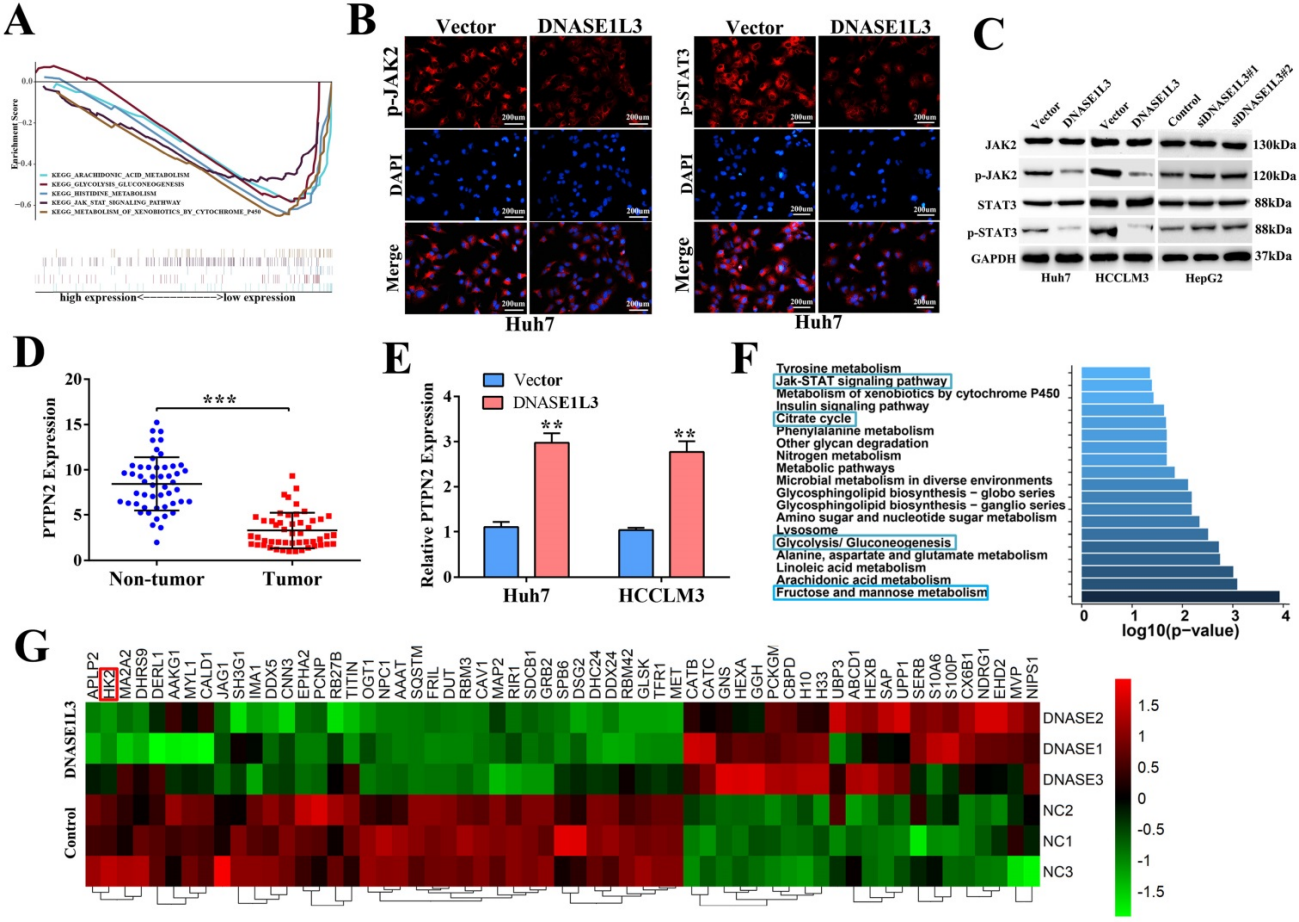
** DNASE1L3 could inhibit the JAK-STAT and glucose metabolism pathways in HCC cells. (A)** Five significantly differential pathways in single-gene GSEA enrichment analysis of DNASE1L3.** (B)** Immunofluorescence staining of p-JAK2 and p-STAT3 in DNASE1L3-overexpressing Huh7 cells and control cells. **(C)** Western blotting was carried out to determine p-JAK2 and p-STAT3 protein expression in DNASE1L3-overexpressing Huh7 and HCCLM3 cells as well as DNASE1L3 knock-down HepG2 cells. **(D)** RT-qPCR was used to investigate PTPN2 expression across 50 pairs of HCC and adjacent non-tumor tissues. **(E)** RT-qPCR allowed for investigation of the mRNA levels of PTPN2 in DNASE1L3-overexpressing Huh7 and HCCLM3 cells.** (F)** Top 20 pathways for proteomics KEGG pathway analysis. *p < 0.05, **p < 0.01, ***p < 0.001.

To better understand the molecular mechanisms involved in the inhibitory effect of DNASE1L3 on tumor growth in HCC, the DNASE1L3 mRNA sequence data was downloaded from TCGA-LIHC. Through single-gene GSEA enrichment analysis, we identified five key pathways demonstrating significant differences which included JAK/STAT and glycolysis/gluconeogenesis signal pathways (Fig. 5A). We first investigated the effect of DNASE1L3 on expression of vital JAK/STAT pathway-realted proteins, including JAK2 and STAT3 in DNASE1L3 overexpressing and knock-down models. Immunofluorescence staining demonstrated that DNASE1L3 up-regulation could inhibit p-JAK2 and p-STAT3 protein levels (Fig. 5B and Supplementary Fig 5). Furthermore, western blotting indicated DNASE1L3 overexpression diminished the protein levels of p-JAK2 and p-STAT3 in Huh7 and HCCLM3 cell lines, whereas depletion of DNASE1L3 promoted p-JAK2 and p-STAT3 levels in the HepG2 cell line (Fig. 5C). PTPN2 is a known critical negative modulator of JAK/STAT signaling, and works to dephosphorylate receptor protein tyrosine kinases such as PDGFR, CSF1R, EGFR and INSR 16. PTPN2 also dephosphorylates non-receptor protein tyrosine kinases such as STAT1, STAT3, and STAT6, Src family kinases as well as JAK1, JAK2 and JAK3 17. Then we investigated the relationship between DNASE1L3 and PTPN2 in HCC progression. We found PTPN2 expression was markedly suppressed in HCC samples in contrast to the corresponding surrounding healthy liver tissues (Fig. 5D). PTPN2 was upregulated in DNASE1L3-overexprssing Huh7 and HCCLM3 cell lines (Fig. 5E). These results suggested that DNASE1L3 might suppress the JAK/STAT signaling pathway by promoting PTPN2 expression.

To further validate the above-mentioned bioinformatics prediction, a label-free proteomics quantitative analysis was performed using the DNASE1L3 overexpressing HCCLM3 model. The result identified 113 differentially expressed proteins (fold change ≥ 2), which included 46 up-regulated and 67 down-regulated proteins (Supplementary Fig. 6). The KEGG pathway enrichment analysis showed that DNASE1L3 could not only inhibit the JAK/STAT signal pathway but could also influence the glucose metabolism of HCC cells (Fig. 5F). Among partially significant differential proteins shown in the heatmap, HK2 was closely linked to glycolysis, and its protein levels were down-regulated when DNASE1L3 was overexpressed (Fig. 5G). These data implied that the inhibitory effect of DNASE1L3 on HCC cells was closely related to the JAK/STAT and glycolysis pathways.

### DNASE1L3 weakened glycolysis via inactivating rate-limiting enzymes involved in the PTPN2-HK2 and CEBPβ-p53-PFK1 pathways

**Figure 6:**
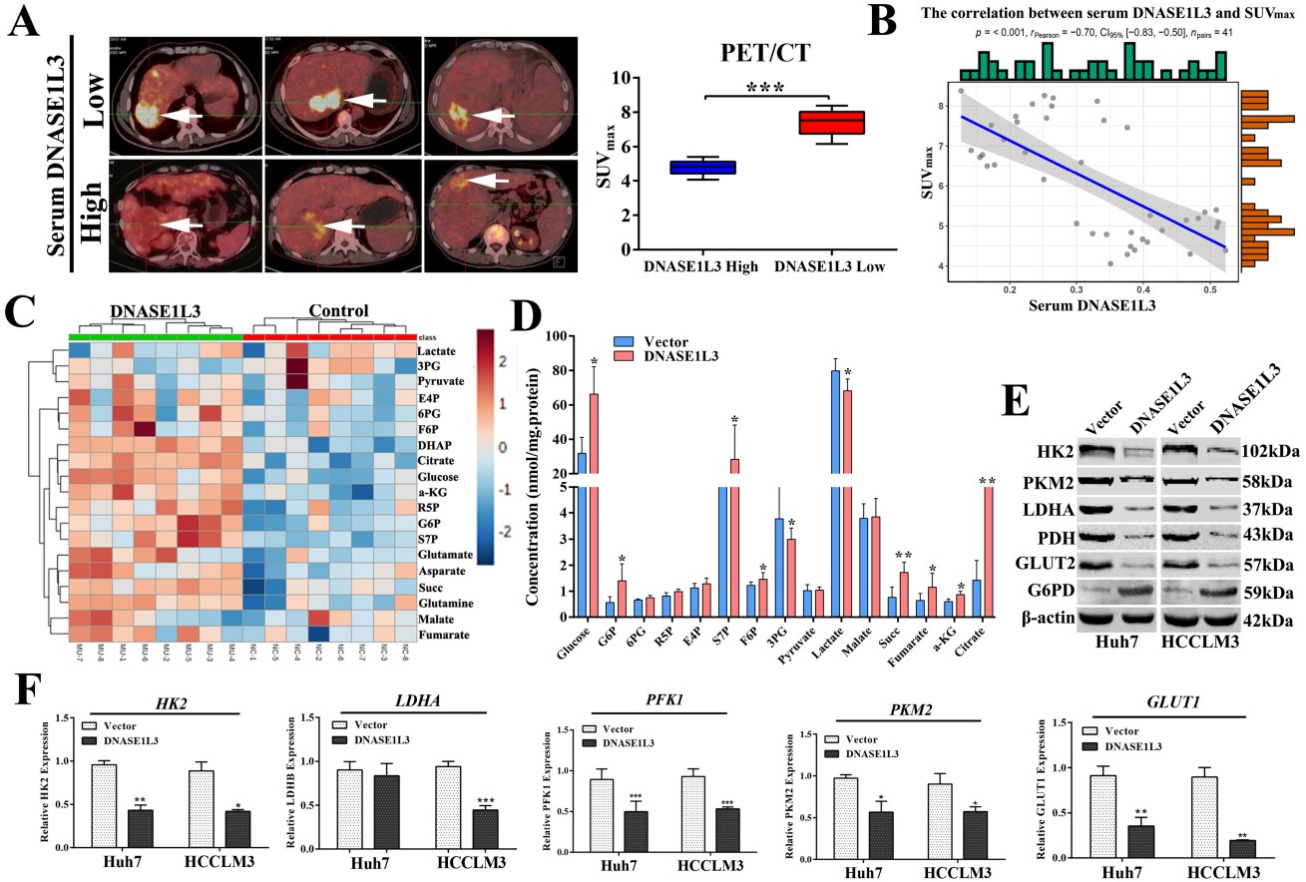
** DNASE1L3 weakened glycolysis through inhibiting rate-limiting enzymes involved in glucose metabolism. (A)** ^18^F-FDG PET/CT and DNASE1L3 ELISA assay were used to investigate the correlation between serum DNASE1L3 activity and glucose metabolism. Representative ^18^F-FDG PET/CT imaging of HCC patients with low or high serum DNASE1L3 levels are depicted (left panel). SUV_max_ analyses of high and low serum DNASE1L3 level groups (n = 41; *p* < 0.001). (right panel). **(B)** The correlation between PET/CT SUV_max_ and serum DNASE1L3 level. **(C)** Label-free quantitative proteomics was performed to assess the metabolites linked to the TCA cycle and glycolysis in DNASE1L3-overexpressing HCCLM3 cells and control cells. **(D)** Metabolite quantification was carried out with liquid chromatography-tandem mass spectrometry (LC-MS/MS). **(E)** Western blotting was performed to evaluate the effects of DNASE1L3 overexpression on protein levels of some key rate-limiting enzymes in glycolysis in Huh7 and HCCLM3 cells.** (F)** RT-qPCR was performed to evaluate the effects of DNASE1L3 overexpression on mRNA levels of some key rate-limiting enzymes in glycolysis in Huh7 and HCCLM3 cells.

To clarify the relationship between DNASE1L3 activity and glucose metabolism in HCC, PET/CT (fluoro-2-D-deoxyglucose F18 ([18F]-FDG) imaging and the serum DNASE1L3 activity of 41 patients with HCC were used to assess the role of DNASE1L3 in glucose metabolism. The results showed that the PET/CT SUVmax of liver tumors in patients with low serum DNASE1L3 was strikingly higher than the HCC patients with elevated serum DNASE1L3 (Fig. 6A). Moreover, the serum DNASE1L3 levels had a significantly negative correlation with PECT/CT SUVmax (Fig. 6B). Next, we further assessed the metabolites related to glycolysis and the tricarboxylic acid (TCA) cycle in DNASE1L3 stably-overexprssing HCCLM3 cells and control HCCLM3 cells using LC-MS/MS. We found DNASE1L3 overexpression reduced glycolytic metabolites, such as glyceraldehyde 3-phosphate and lactic acid, whereas it increased intermediate products of the Krebs cycle including citric acid, α-ketoglutarate, succinic acid, and fumaric acid (Fig. 6C and D). These results strongly implied that DNASE1L3 inhibited glycolysis in HCC. In addition, the correlation analysis of central carbon metabolism and proteomics in DNASE1L3 stably-overexpressing HCCLM3 cells confirmed the extensive involvement of proteomic differential proteins in the production of five metabolites (Supplementary Fig. 7).

To further explore the mechanism underlying the role of DNASE1L3 on reprogramming glucose metabolism, we examined the effect of DNASE1L3 on the mRNA and protein expression of some rate-limiting enzymes in glycolysis in HCC cells. The results demonstrated that overexpression of DNASE1L3 could dramatically decrease the protein and mRNA levels of HK2, LDHA, PKM2, and PFK1 (Fig. 6E and F).

**Figure 7:**
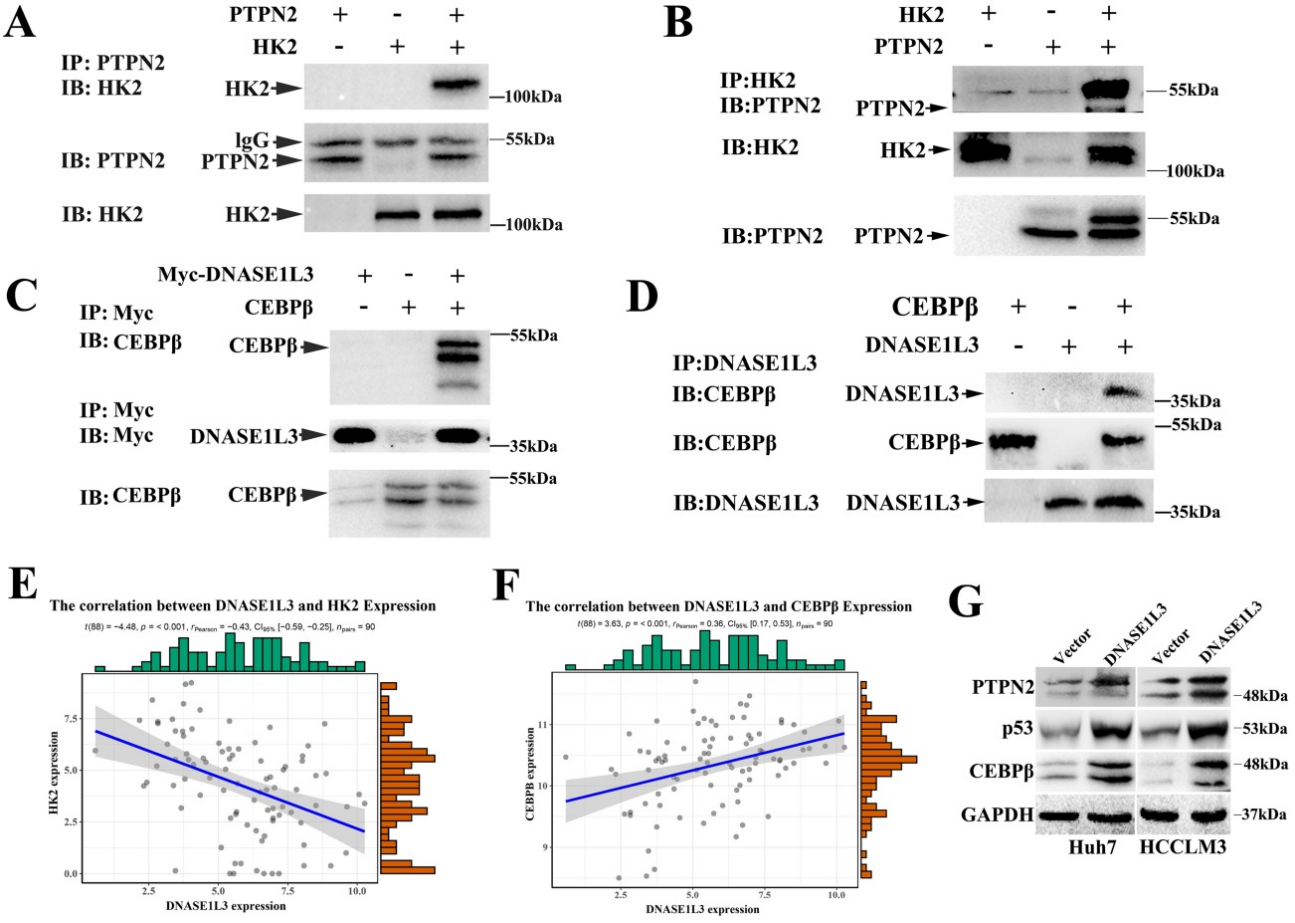
** DNASE1L3 inhibited glycolysis via suppressing PTPN2-HK2 and CEBPβ-p53-PFK1 pathways. (A-B)** Co-IP assays were conducted to assess the direct interaction of HK2 and PTPN2 in Huh7 cells.** (C-D)** Reciprocal co-IP assays were conducted to determine DNASE1L3 and CEBPβ interactions in Huh7 cells transfected with myc-DNASES1L3 and CEBPβ.** (E-F)** The correlation between DNASE1L3 expression and HK2 as well as CEBPβ. **(G)** PTPN2, p53, and CEBPβ protein levels in DNASE1L3 overexpressing Huh7 and HCCLM3 cells. *p < 0.05, **p < 0.01, **p < 0.001.

As a rate-limiting enzyme in the first step of glycolysis, HK2 played a crucial role in glucose metabolism. Previous research had proved that c-Src could interact with and phosphorylate human HK1 at Tyr 732 and HK2 at Tyr 686, which was essential for HK1 and HK2 to catalyze the conversion of glucose to glucose-6-phosphate, the committed step of glycolysis^18^. As a key regulatory enzyme of c-Src, PTPN2 could dephosphorylate Src family kinases. However, PTPN2 induces or inhibits the expression of HK2 by dephosphorylating c-Src is still unclear. Expectedly, we observed a negative correlation between the expression of DNASE1L3 and HK2 in GSE15765 datasets (Fig. 7E) and overexpression of DNASE1L3 could increase PTPN2 protein levels (Fig. 7G), so we speculated that DNASE1L3 inhibited the expression of HK2, possibly due to the interaction between PTPN2 and HK2. Therefore, we performed a co-IP assay to explore the interaction between PTPN2 and HK2. The results indicated that HK2 was specifically precipitated by PTPN2. This interaction was further confirmed by reciprocal co-IP assay using overexpressed HK2 and PTPN2 (Fig. 7A and B).

PFK1 is an important regulatory gene involved in the p53-mediated aerobic and glycolytic pathways. Moreover, we had confirmed PFK1 as a downstream target gene of DNASE1L3. Protein mass spectrometry suggested DNASE1L3 could interact with CEBPβ, and the expression between DNASE1L3 and CEBPβ demonstrated a positive relationship in GSE15765 datasets (Fig. 7F). Based on these findings, we speculated that the CEBPβ-p53-TIGAR-PFK1 pathway could play a role in the inhibitory effect of DNASE1L3 on glycolysis by the interaction between DNASE1L3 and CEBPβ in HCC. Subsequently, co-IP assays were performed to substantiate this interaction in overexpressed myc-DNASE1L3 and CEBPβ cell lines (Fig. 7C and D). The overexpression of DNASE1L3 resulted in the upregulation of protein levels of CEBPβ and p53 (Fig. 7G). CEBPβ is an upstream transcription factor of p53, which promotes p53 expression. P53 can directly transcribe and activate TIGAR (TP53-induced glycolysis and apoptosis regulator). TIGAR-encoded protein can degrade fructose 2,6-diphosphate, which is the strongest activator of the key enzyme PFK1 in the glycolytic pathway of tumor cells^19^. These results suggested that DNASE1L3 mediated the inhibitory glycolytic pathway involved in the CEBPβ-p53-PFK1 pathway.

### Inhibition of DNASE1L3 function by ZNF384 was promoted by hepatitis B virus X protein in HCC

**Figure 8:**
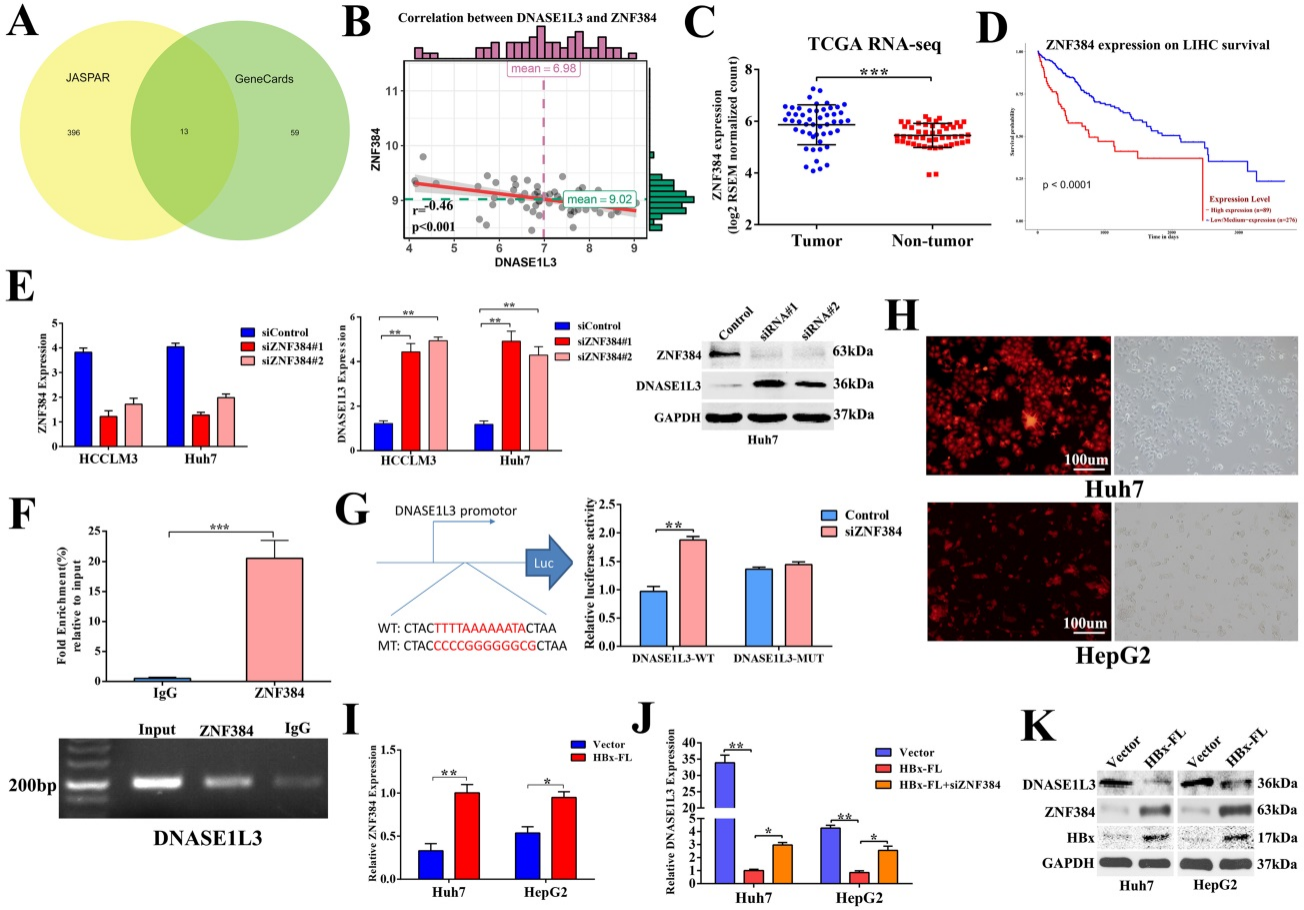
** ZNF384 could negatively regulate the DNASE1L3 expression in human HCC. (A)** Thirteen potential transcriptional factors were identified via intersecting JASPAR and GeneCards databases. **(B)** The TCGA-LIHC dataset was used to analyze the correlation between DNASE1L3 and ZNF384 expressions. **(C)** ZNF384 expression in 50 pairs of HCC and adjacent healthy tissue samples were downloaded from the TCGA-LIHC dataset. **(D)** Kaplan-Meier analysis of overall survival based on ZNF384 expression in 364 patients with HCC downloaded from the TCGA-LIHC dataset. **(E)** RT-qPCR and western blotting were conducted to analyze the impact of decreased ZNF384 on DNASE1L3 expressions in Huh7 and HCCLM3 cells using siRNA. **(F)** ChIP assays with normal IgG or anti-ZNF384 were performed to recognize ZNF384 binding sites on the DNASE1L3 promoter in Huh7 cells. **(G)** Luciferase reporter assay was used to demonstrate the effect of siRNA-mediated knockdown of ZNF384 on the promoter activity of wild type and mutant DNASE1L3.** (H)** HBx stably expressed HepG2, Huh7 and lentiviral vector expressed red fluorescent protein.** (I)** RT-qPCR was carried out in order to evaluate the effect of HBx overexpression on ZNF384 mRNA levels **(J)** RT-qPCR was performed to examine the relative DNASE1L3 expression after Huh7 and HepG2 cell lines transfection with siZNF384 which stably overexpressed HBx. **(K)** Western blotting was performed to assess the effect of HBx overexpression on protein levels of ZNF384 and DNASE1L3. *p < 0.05, **p < 0.01, **p < 0.001.

To further explore the mechanism underlying the down-regulation of DNASE1L3 in HCC, we first predicted the transcriptional factors of DNASE1L3 using JASPAR and GeneCards. Next, we intersected the outcomes of the two databases. Thirteen potential transcriptional factors with binding sites in the 2-kb region upstream from the transcription start site of DNASE1L3 were identified (Fig. 8A). An analysis of the correlation of the mRNA levels between DNASE1L3 and 13 transcriptional factors using the RNA-seq data from TCGA-LIHC (Supplementary Table 4) revealed that DNASE1L3 was negatively regulated by its upstream transcriptional factor zinc-finger protein 384 (ZNF384) (Fig. 8B). Next, Through the TCGA database, we accessed the mRNA levels of ZNF384 and the survival time in HCC and normal tissues. The results indicated that ZNF384 was upregulated in HCC tissues compared with adjacent normal tissues (Fig. 8C). The Kaplan-Meier analysis demonstrated that the high expression of ZNF384 was significantly associated with shorter overall survival time (Fig. 8D). Further mechanism analysis revealed that the copy number variations (CNVs) could influence the mRNA levels of ZNF384 (Supplementary Fig. 8). To verify whether ZNF384 was involved in regulating the expression of DNASE1L3, we performed an *in vitro* loss-of functional study to evaluate the effect of ZNF384 on the expression of DNASE1L3 in HCC cells. We found the DNASE1L3 mRNA and protein levels were increased in ZNF384 knock-down Huh7 and HCCLM3 cells (Fig. 8E). To further dig out the molecular mechanisms of ZNF384-dependent DNASE1L3 down-regulation, we performed CHIP-PCR and luciferase reporter assays. The results showed that ZNF384 could directly bind to the DNASE1L3 promoter region located between +437 and +449 from the transcription start site (Fig. 8F). The knockdown of the ZNF384 gene stimulated the promoter activity of the DNASE1L3 gene, whereas a mutation in the nucleotides in the presumed targeting sites of DNASE1L3 led to complete annulment of the positive effect (Fig. 8G). These findings concluded that ZNF384 could suppress the expression of DNASE1L3 in human HCC.

Because chronic hepatitis B virus (HBV) infection is the leading cause of HCC 20 and the X protein of HBV (HBx) is an imperative regulator of HBV, we next determined if HBx regulated the expression of DNASE1L3 via ZNF384. In HBx stably-overexpressed Huh7 and HepG2 cell lines (Fig. 8H), we found increased expression of ZNF384, whereas DNASE1L3 remarkably declined (Fig. 8I-K). However, knock-down of ZNF384 could partially reverse the down-expression of DNASE1L3 induced by HBx (Fig. 8J). These results indicated that HBx could suppress the expression of DNASE1L3 by upregulation of ZNF384.

## Discussion

A key mediator of chronic inflammatory liver diseases and hepatocarcinogenesis is hepatocyte death. Targeting of apoptosis is a promising avenue of HCC treatment that might yield novel treatment strategies for this deadly inflammation-driven cancer 21. DNASE1L3 could function as the backup endonuclease for caspase-activated DNase (CAD) in the secondary necrosis phase of apoptosis both *in vivo* and *in vitro* 21. Previous studies have reported that DNASE1L3 meets most of the biochemical and functional criteria (i.e., cation dependence, pH profile, ability to cleave single strand (ss) and double-strand (ds) DNA with 3' -OH ends, and nuclear localization) expected of an enzyme involved in apoptosis 14. Therefore, DNASE1L3 was once regarded as one of the most promising personalized gene-based therapy for melanoma, as well as for other malignancies 22. In the present study, we used a multi-step strategy to analyze DEGs from HCC datasets and identify prognostic gene signatures in HCC. We found DNASE1L3 was the only DNase member among these DEGs that was significantly down-regulated in HCC tissues and cell lines. Moreover, its expression was tightly associated with tumor grades of HCC. More importantly, univariate and multivariate analyses revealed that the low expression of DNASE1L3 that predicted poor prognosis was also an independent risk factor for poor prognosis of patients with HCC, suggesting its tumor-inhibiting effect. These results are supported by published literature 20.

However, the concept of DNASE1L3 being involved in intra-nuclear or intracellular DNA fragmentation or during the process of apoptosis has not been sufficiently confirmed or proven by any experiment until now. Since there is evidence to suggest that DNase1 activity is inhibited in the cytoplasm and nucleus by actin, which is abundant in both locations and serves as the major DNase1 inhibitor. As the major extracellular nuclease, the delivery of DNase1 gene into the cell is not expected to trigger apoptosis because it is not a component of the cascade, and overexpressed DNase1 would be denied access to the nucleus owing to a lack of a nuclear localization signal (NLS) 22. However, in the current study,* in vitro* functional assays revealed DNASE1L3 repressed the HCC cell proliferation, migration, and invasion, which probably partially arrested the cell cycle and promoted cell apoptosis. *In vivo* assays also supported the tumor-inhibiting function. We observed DNASE1L3 significantly reduced the protein levels of c-myc, Bcl2, and CyclinD1, while increased the protein levels of Bax and cleaved-caspase 3. It is well-known that the pro-apoptotic BCL-2 proteins, BCL-2-associated X protein (BAX), causing the release of intermembrane space proteins, such as cytochrome c, into the cytoplasm to activate the caspase cascade 23. Our findings support the notion that DNAS1L3 endonuclease activity is not only important for the degradation of genomic DNA as a terminal apoptotic step but rather is a participating factor in the overall process of cell death 24.

Metabolism regulates the apoptotic machinery both directly or indirectly, and cancer cells utilize the altered metabolism to evade apoptosis 25. Indeed, cancer cells prefer aerobic glycolysis to oxidative phosphorylation even under normoxic conditions, a characteristic known as the Warburg effect. It is widely accepted that the “Warburg effect” is part of the fundamental metabolic changes that cancer cells undergo to develop the propensity to grow and proliferate uncontrollably as well as to inhibit apoptosis 26. Hence, there exists an urgent need to develop a novel therapeutic strategy that targets the altered metabolic traits to selectively eliminate cancer cells by promoting apoptosis. In our study, we identified that DNASE1L3 could not only inhibit tumor cell proliferation, migration, and invasiveness by inducing G0/G1 cell cycle arrest and cell apoptosis but also weakened glycolysis via inactivating rate-limiting enzymes involved in the PTPN2-HK2 and CEBPβ-p53-TIGAR-PFK1 pathways (Supplementary Fig 9). This finding ascribes a novel function of DNASE1L3 as an inhibitor of glycolysis to synergistically induce apoptosis in HCC cells.

The underlying mechanisms of DNASE1L3 function in cancer development and progression have not been clarified until now. Here, we used a combined approach of metabolomics and proteomics that revealed the inhibitory effect of DNASE1L3 on tumor cells to be closely related to the JAK/STAT and glycolysis pathways. Multiple lines of evidence suggest the JAK/STAT pathway is a promising therapeutic target in HCC 27, 28. Similarly, we have shown in our study that DNASE1L3 could suppress the JAK/STAT signaling pathway by promoting the expression of PTPN2. To further investigate the effect of DNASE1L3 on glycolysis pathways, we explored the relationship between serum DNASE1L3 activity and cancer glycometabolism reprogramming in patients with HCC. The use of 18F-FDG to detect tumors with an elevated glucose uptake in current clinical applications is based on the manifestation of the Warburg effect. Using the 18F-FDG PET/CT scanning and serum DNASE1L3 activity analysis, we verified that DNASE1L3 was strongly related to glycolysis in HCC. More studies demonstrated DNASE1L3 weakened glycolysis via inactivating rate-limiting enzymes involved in the PTPN2-HK2 and CEBPβ-p53-TIGAR-PFK1 pathways. HK2 was a rate-limiting enzyme in the first step of glycolysis. Previous studies reported that c-Src could interact with and phosphorylate human HK2 at Tyr 686, which is essential for HK2 to catalyze the conversion of glucose to glucose-6-phosphate, the committed step of glycolysis^18^. Until now, the interaction between PTPN2 and HK2 has not been well determined. An earlier study showed that PTPN2 could dephosphorylate Src family kinases; however, whether it could induce or inhibit the expression of HK2 by dephosphorylating c-Src is still unclear. Using reciprocal co-IP assays, we first confirmed that PTPN2 interacted with HK2, implying that the JAK/STAT pathway could affect the glycolysis pathway by PTPN2-HK2. As a rate-limiting enzyme of glycolysis, PFK1 is an important regulatory gene involved in p53-mediated aerobic and glycolytic pathways 29. We confirmed DNASE1L3 could interact with CEBPβ to promote the expression of p53 expression further to activate TIGAR, the strongest activator of the key enzyme PFK1 in the glycolytic pathway of tumor cells.

ZNF384 encodes a C2H2-type zinc finger protein that functions as a transcription factor and regulates the transcription of the extracellular matrix genes. Previous studies have demonstrated that ZNF384 regulates MMP1, MMP3, and COL1A1 gene transcription 30. Here, we explored the mechanism underlying the down-regulation of DNASE1L3 in HCC and found DNASE1L3 is a target of ZNF384. We verified that ZNF384 could directly bind to the DNASE1L3 promoter region. The HBx is a non-structural protein that plays an important role in hepatocytes, promoting the progression of liver disease in patients infected with HBV. We further found that HBx could suppress the expression of DNASE1L3 by promoting the expression of ZNF384.

## Conclusions

We investigated the effects of DNASE1L3 on HCC growth, apoptosis, and glucose metabolism reprogramming. We demonstrated the down-regulation of DNASE1L3 was a critical feature of HCC development. Furthermore, we confirmed DNASE1L3 was an important tumor-suppressor gene as it participated in cell apoptosis and inhibits glycolysis involved in inactivating rate-limiting enzymes. We believe our findings would open new avenues of research into uncovering the link between apoptotic defects and unique metabolic preferences in HCC, which would help us developing a novel therapeutic strategy targeting the altered glucose metabolism reprogramming trait and simultaneously promoting apoptosis.

## Supplementary Material

Supplementary materials and methods, figures, and tables.
