# Supplementary material for: Deoxyribonuclease 1-like 3 Inhibits Hepatocellular Carcinoma Progression by Inducing Apoptosis and Reprogramming Glucose Metabolism
Source: Int J Biol Sci. 2022 Jan 1;18(1):82–95. doi: 10.7150/ijbs.57919 (PMC8692146; doi:10.7150/ijbs.57919)
Supplement: Supplementary file 1 — Supplementary materials and methods, figures, and tables. [file ijbsv18p0082s1.pdf]

Deoxyribonuclease 1-like 3 Inhibits Hepatocellular Carcinoma Progression by Inducing Apoptosis and Reprogramming Glucose Metabolism

Yusha Xiao[a,b], Kang Yang [c], Pengpeng Liu[b], Dong Ma[b], Ping Lei[d,]*, Quanyan Liu[e,]*

[a] Department of Cardiovascular Surgery, Zhongnan Hospital of Wuhan University, Wuhan 430071, P.R. China.

[b] Department of Hepatobiliary Surgery, Zhongnan Hospital of Wuhan University, Wuhan 430071, P.R. China.

[c] Department of Urology, Renmin Hospital of Wuhan University, Wuhan 430060, China.

[d]Department of Geriatrics, Tianjin Medical University General Hospital Hospital, Tianjin, 300052, P.R. China.

[e] Department of Hepatobiliary Surgery, Tianjin Medical University General Hospital , Tianjin, 300052, P.R. China.

*Corresponding authors: Quanyan Liu. Tel.: +86-22-60362760; Fax: +86-22-60362670, E-mail: spsslqy@vip.126.com; Ping Lei. Tel: +86-22-60362762; Email: leiping1974@163.com.

Supplementary information

Materials and methods

Bioinformatics

Microarray data were downloaded from the Gene Expression Omnibus database (accession numbers GSE76427, GSE64041, GSE57957, GSE45436), and R software (https://bioconductor.org/biocLite.R) was used to analyze the relative expression level of DNASE1L3. The RNA-seq data of HCC and Kaplan-Meier plots analyzing the survival of HCC patients were obtained from The Cancer Genome Atlas (TCGA) liver cancer dataset (LIHC) from UCSC Xena (https://xenabrowser.net/). All differentially expressed genes were enriched based on Gene set enrichment analysis (GSEA) (www.broadinstitute.org/gsea) to identify the significant pathways. Prediction of DNASE1L3 promoter binding regions was conducted via GeneCards (https://www.genecards.org) and Jaspar (http://jaspar.genereg.-net/).

Cell culture and reagent

Human HCC cell lines used for this study include Huh7, SK-hep1, HepG2, Hep3B, HCCLM3 and immortalized human liver cell line of HL–7702 (L02). The cell lines were obtained from the Cell Bank of Type Culture Collection (CBTCC, the Chinese Academy of Sciences, Shanghai, China) and characterized by mycoplasma testing, DNA fingerprinting, isozyme detection, and cell viability determination by third-party biology

services (GeneCreate Biological Engineering Co., Ltd, Wuhan, China). Those cell lines were cultured in high-glucose DMEM (Gibco, USA) supplemented with 10% fetal bovine serum (Gibco, USA). All cells were cultured in an incubator at 37 °C with 5% CO_2.

Patient and clinical sample collection

A retrospective cohort (Cohort I) of 80 HCC patients who had undergone routine surgical procedures at the Zhongnan Hospital of Wuhan University, Wuhan, China, was included in the present study. Ethical approval was obtained from the Ethics Committee of the Zhongnan Hospital of Wuhan University, and informed consent was obtained from each patient. Those specimens were stored at −80 °C in an RNA stabilization solution immediately. The pathological diagnosis of HCC was performed according to the World Health Organization (WHO) criteria. The clinical data of patients were obtained from the electronic medical record of the general surgery department of Zhongnan Hospital of Wuhan University. The use of all samples was approved by the Committee for Ethical Review of Research at the Zhongnan Hospital of Wuhan University, Wuhan, China. Inclusion criteria were as follows: primary diagnosis of HCC between 2005 and 2014 (at least five years of potential follow-up); no previous diagnosis of carcinoma; and no evidence of disease within one month of primary surgery. Patients who received neoadjuvant treatment before primary surgery were excluded.

All patients were routinely examined every 3–6 months during the first five years of follow-up and once a year thereafter. The overall survival time was defined as the period in months from operation to death. The disease-free survival time was defined as the period in months from operation to the date at which relapse was clinically identified.

Cohort II consisted of 72 HCC patients who underwent preoperative DNASE1L3 ELISA assay between January 2018 and December 2020 at the Zhongnan Hospital of Wuhan University, Wuhan, China, and 54 healthy people. The pathological diagnosis of HCC was performed according to the WHO criteria. None of the patients received preoperative chemotherapy or radiotherapy. The use of all samples was approved by the Ethics Committee of the Zhongnan Hospital of Wuhan University, Wuhan, China. The clinical data of patients were used to analyze the difference in serum DNASE1L3 activity between HCC patients and healthy people. Of the 72 patients in this cohort, 41 HCC patients underwent preoperative DNASE1L3 ELISA Assay and then positron emission tomography (PET)/computed tomography (CT) examination. These patients' data were used to analyze the correction between glucose metabolism and serum DNASE1L3 activity.

Total RNA isolation and quantitative real-time PCR (qRT-PCR)

TRIzol Reagent was used to separate total RNA from tumor tissues and cells. Reverse transcription of mRNA was conducted with PrimeScript

RT Master Mix (Takara, Japan). Quantitative real-time PCR was performed using SYBR qPCR Mix (Toyobo, Osaka, Japan) and a CFX Connect Real-Time PCR Detection System (Bio-Rad, USA). The relative gene expression was measured using the $2^{-\Delta\Delta CT}$ method. The GAPDH was used as an internal control.

Plasmid construction, lentiviral construction, RNA interference, and cell transfections

DNASE1L3 was inserted into the pcDNA3.1 vector purchased from GeneCreate (Wuhan, China) and transfected into Huh7 and HCCLM3 cells using Lipofectamine® 3000 reagent (Thermo Fisher's Scientific, Waltham, USA). Lentiviral vectors and control plasmids were synthesized and purchased from GeneCopoeia (Guangzhou, China), and we established DNASE1L3-stably overexpressed HCCLM3 cells. Two pairs of siRNAs (siRNA#1-#2) were designed to knock down DNASE1L3 were bought from GeneCreate (Wuhan, China). The siRNA of ZNF384 was designed and purchased from GeneCopoeia (Guangzhou, China). Cells were transfected with 20 nM siRNA using LipoJet reagent (SignaGen, Rockville, MD, USA) following the manufacturer's protocol. Lentivirus carrying full-length HBx (HBx-FL) and lentiviral particles for negative control were constructed by GeneCopoeia (Wuhan, China) and stably transfected into Huh7 and HepG2 cell lines. The siRNA sequences applied in this study and primers designed for PCR of full-length HBx are

provided in Supplementary Table 1.

Immunohistochemistry (IHC) and immunofluorescence (IF)

The tumor tissues were collected and fixed with 4% paraformaldehyde, embedded in paraffin, and sectioned to 4 μm thickness. Then, the tissue sections were exposed to the antigen by incubation with dewaxed water and at 100 °C for 15 min. The tissues were incubated with 3% H_2O_2 for 10 min at 37 °C to block endogenous peroxidase activity, following incubation with BSA and primary antibody overnight at 4 °C. After incubation with HRP-labeled secondary antibodies, HRP was detected with standard substrates for subsequent detection. The sections were stained with hematoxylin and dehydrated with graded alcohol and xylene.

For immunofluorescence, the cells were washed twice and fixed with 4% paraformaldehyde, then permeabilized with 0.5% Triton X–100, then the cells were incubated with primary and secondary antibodies according to the manufacturer's protocol. The coverslips were counterstained with DAPI and imaged with Olympus FV1000 (Tokyo, Japan).

Cell proliferation assay

After cells were transfected for 48 h with the desired siRNA or plasmid, cell proliferation was measured using CCK8 assay according to the manufacturer's instruction. For colony formation assay, the cells transfected after 48 h were seeded (1000/well) in 6-well plates for 7–10 days. Colonies (≥50 cells/colony) were calculated after fixation with 4%

paraformaldehyde and stained with crystal violet solution.

Cell migration and invasion assay

Cell migration and invasion were measured using the scratch assay. After transfection with desired plasmid or siRNA, about 10^6 cells were seeded into 6-well plates under serum-free conditions and the scratches were made via a 100 µl plastic pipette tip. The migration ability was evaluated 24 and 48 h later by measuring the migration distance. The ability of invasion was assessed using a Matrigel chamber (BD Biosciences, NJ, USA). The transfected cells (6×10^4) were scattered in the upper chamber evenly under serum-free conditions, and culture medium containing 10% FBS was added into the lower chamber for the chemokines to induce cell migration. The migrated cells on the surface were fixed and stained with crystal violet and counted.

Flow cytometry analysis

Cells were transfected with siRNA sequence or plasmid before stained with AnnexinV-FITC and propidium iodide (PI). Subsequently, FACSCalibur flow cytometer (BD Biosciences, USA) was used to analyze the cells. For cell cycle analysis, the cells were stained with cell cycle staining kit (BD Biosciences, USA).

Western blotting

Protein samples were prepared using RIPA cell lysis buffer with protease inhibitors and phosphatase inhibitors (Roche, Mannheim, Germany). The

total protein quantity was measured using the BCA assay. The samples were separated via 10% SDS-PAGE and were transferred onto PVDF membranes, and incubated at 4 °C overnight with primary antibodies against the indicated proteins on a rotating wheel. After incubation with HRP-conjugated secondary antibodies for 1 h, antibody-binding proteins were detected using Clarity™ Western ECL Substrate (Bio-Rad, Hercules, CA, USA). The information on antibodies used in our study is provided in Supplementary Table 2.

Chromatin immunoprecipitation assay

In order to detect the interaction between the DNA and proteins, chromatin immunoprecipitation (ChIP) assay was performed using the Magna ChIP-seq™ Chromatin Immunoprecipitation Kit (Millipore; Billerica, USA) according to manufacturer's instructions. ZNF384 or IgG antibody and protein A/G beads were incubated overnight at 4 °C. Afterward, DNA was extracted from the DNA–protein complex. The immunoprecipitation DNA was verified by ChIP-PCR.

Tumorigenesis assay

Five-week-old male BALB/c nude mice were purchased from the Animal Center of the Chinese Academy of Medical Sciences (Beijing, China). Guidelines for Care and Use of Laboratory Animals were followed during the investigation. All animal procedures were approved by the Ethics Committee of the Zhongnan Hospital of Wuhan University. All animals

were maintained under specific pathogen-free conditions. For tumor growth analysis, HCCLM3 cells (1×10^6/100 μL PBS) that stably overexpressed DNASEL13 or control were subcutaneously injected into the armpits of six nude mice per group. The tumor volume was calculated every five days. The mice were sacrificed six weeks after injection, and the tumors were dissected and imaged. To verify the intrahepatic antitumor effect, an orthotopic HCC mouse model was established. First, the subcutaneous tumors of mice were implanted based on the protocols mentioned above. Then tumors were collected when they reach 1 cm in diameter and cut into pieces of about 1 mm^3 in size under aseptic conditions. After washing with PBS, the tumors were waterlogged in the serum-free medium. Afterward, the nude mice were anesthetized, and then a midline abdominal incision was made to expose the liver, subsequent one piece of tumor was implanted into the left liver lobe of each mouse. Finally, a suture compression was done to stop bleeding, and the abdominal incision was sutured. After six weeks, the mice were sacrificed to dissect liver tumors for the following experiments.

Label-free quantitative proteomics

Label-free quantitative proteomics was carried out by GeneCreate Biological Engineering Co., Ltd. (Wuhan, China). The mass spectrometry data were collected using Q Exactive (Thermo Fischer Scientific, Waltham, MA, USA) and a liquid-mass system. Protein lysate was added

to the cell precipitate and then incubated on ice for 5 min. DTT with a final concentration of 10 mM was added, followed by an ice bath with ultrasound for 15 min, then 13,000 g was centrifuged at 4 °C for 20 min. The supernatant was removed and transferred to a new centrifuge tube. About four volumes of pre-chilled acetone were added into the centrifuge tube, the final concentration of 30 mM DTT, at −20 °C overnight. The protein precipitate was collected by centrifugation and dried in air. The protein was redissolved with 8 M urea/100 mM TEAB (pH 8.0) solution, DTT was added to the final concentration of 10 mM and incubated at 56 °C for 30 min. Then, iodoacetamide was added to the final concentration of 55 mM and placed in the dark for 30 min at room temperature for alkylation. Protein concentrations were determined using the Bradford method. About 100 g of protein was digested by trypsin. After the protein solution was diluted five times with 100 mM TEAB, trypsin was added at a mass ratio of 1:50 (trypsin: protein) and digested overnight at 37 °C. The peptides after enzymatic hydrolysis were desalted with the C18 column, and the peptides after vacuum freeze-drying were desalted.

Metabolite quantification by liquid chromatography-tandem mass spectrometry (LC-MS/MS)

Metabolite quantification was conducted by Biotree Biotech (Shanghai, China). Briefly, 80% HPLC-grade methanol (cooled to −80 °C) was

added to the frozen cells and vortexed for 1 min at 4 °C and incubated for 4 h at −80 °C, and then centrifuged at 14,000 g for 15 min using a refrigerated centrifuge with 4 °C. The supernatant was transferred to a new 1.5 mL tube, and speedy Vac/lyophilize for LC-MS analysis. For quantification of absolute metabolites concentrations, U–1^{3}C-glutamine was used as an internal standard and was added to the extraction buffer containing 80% methanol. All samples and standards were measured using a TSQ VANTAGE interfaced with Ultimate 3000 Liquid Chromatography system (Thermo Fischer Scientific), equipped with a HILIC HPLC carbon column (Amide 4.6 × 100 mm ID 3.5 μm; Part No:186004868, Waters). Mobile phase buffer A (1L) contained 20 mM ammonium hydroxide, 20 mM ammonium acetate, 95% water and 5% (v/v) acetonitrile, pH 9.0 at 25 °C. While the mobile phase buffer B contained 100% acetonitrile. The column was maintained at a controlled temperature of 30 °C and was equilibrated with 15% buffer A for 3 min at a constant flow rate of 350 μL/min. 10 μL aliquots of each sample were loaded onto the column, and the compounds were eluted from the column with a linear gradient of 15–50% buffer A from the 4th to 16th min, and then increased to 85% buffer A from 16th min to 20th min and decreased to 15% buffer A from 21th to 23th min, and the column was washed for 2 min with 15% Buffer A. The ion transfer tube temperature was set to 350 °C and vaporizer temperature was 270 °C. The instrument

was run in negative mode with a spray voltage of 3000 sheath gas 40 and Aux gas 5.0. About seven to eight concentrations (from low to high) of the different standard mixtures were measured using multiple reactions monitoring mode (MRM) with optimal collision energies to produce a standard curve.

Hematoxylin-eosin (H&E) staining

The collected tumor samples from the mice liver were fixed in 4% paraformaldehyde. The tissues were dehydrated, embedded in paraffin, and cut into slices. The sections were stained with hematoxylin and eosin and observed by light microscopy (Olympus, Tokyo, Japan).

DNASE1L3 Elisa Assay

Natural coagulation of blood was performed by centrifuging the blood sample at 2000–3000 rpm for 10–20 min at room temperature. The purified DNASE1L3 antibody was used to coat the microporous plate. Standard materials and serum samples to be tested were added to the microporous plate in turn and then combined with the HRP-labeled DNASE1L3 antibody to form the antibody-antigen-enzyme labeled antibody complex. After thorough washing, the substrate TMB was added for color development. TMB turns blue after reacting with the HRP enzyme and then into yellow after the addition of hydrochloric acid. The color intensity was positively correlated with DNASE1L3 in the sample. The absorbance value (O.D. value) was measured with an enzyme marker

at 450 nm, and the concentration of DNASE1L3 in the sample was calculated using a standard curve.

Dual-luciferase reporter activity assay

Indicated sites of DNASE1L3 promoter and corresponding mutant plasmids were cloned into the PGL3.0 luciferase reporter vector and co-transfected with siRNA of ZNF384 into Huh7 cells. Luciferase activity was measured using a Dual-Luciferase Assay Kit (Promega; Madison, WI, USA) 48 h later according to the manufacturer's instructions.

Supplementary Figure Legends

Supplementary Fig. 1. Integrated analysis of HCC datasets recognized 21 significantly differentially expressed genes. (A) 21 mRNAs were identified using the Venn diagram. **(B)** The mRNA expression of 21 genes in TCGA-LIHC datasets.

Supplementary Fig. 2. The expression of DNASE1L3 in different mRNA expression datasets. (A-D) Heatmaps of 21 recognized DEGs in GSE76427, GSE64041, GSE57957, and GSE45436. **(E)** DNASE1L3 expression in 50 pairs of HCC and adjacent non-tumor group downloaded from the TCGA-LIHC dataset. **(F)** Differential expression of DNASE1L3 in HCC grades. **(G-H)** Kaplan–Meier analysis of OS and DFS based on the DNASE1L3 expression in 364 HCC patients downloaded from the

TCGA-LIHC. *$p < 0.05$, **$p < 0.01$, ***$p < 0.001$.

Supplementary Fig. 3. The Kaplan–Meier analysis of Dnase1, Dnase2, and DFFB involved in overall survival (OS) of HCC patients.

Supplementary Fig. 4. The Kaplan-Meier analysis of OS according to DNASE1L3 expression levels in patients with breast cancer, lung adenocarcinoma, pancreatic cancer, sarcoma, melanoma, and kidney cancer.

Supplementary Fig. 5. Immunofluorescence staining of HK2, PKM2, p-JAK2 and p-STAT3 in nude mice HCC tissues.

Supplementary Fig. 6. The heatmap of the identified 113 differentially expressed proteins in label-free proteomics quantitative analysis.

Supplementary Fig. 7. The correlation analysis of central carbon metabolism and proteomics in DNASE1L3 stably-overexpressed HCCLM3 cells.

Supplementary Fig. 8. The correlation between ZNF384 expression and the copy number variation was analyzed in TCGA-LIHC dataset.

Supplementary Fig. 9. Schematic model of the effect of DNASE1L3 on glucose metabolism reprogramming. DNASE1L3 overexpression weakened glycolysis in HCC cells and tissues via inactivating the rate-limiting enzymes involved in PTPN2/JAK/STAT3-HK2 and

CEBPβ-p53-PFK1 pathways.

Supplementary Fig. 1

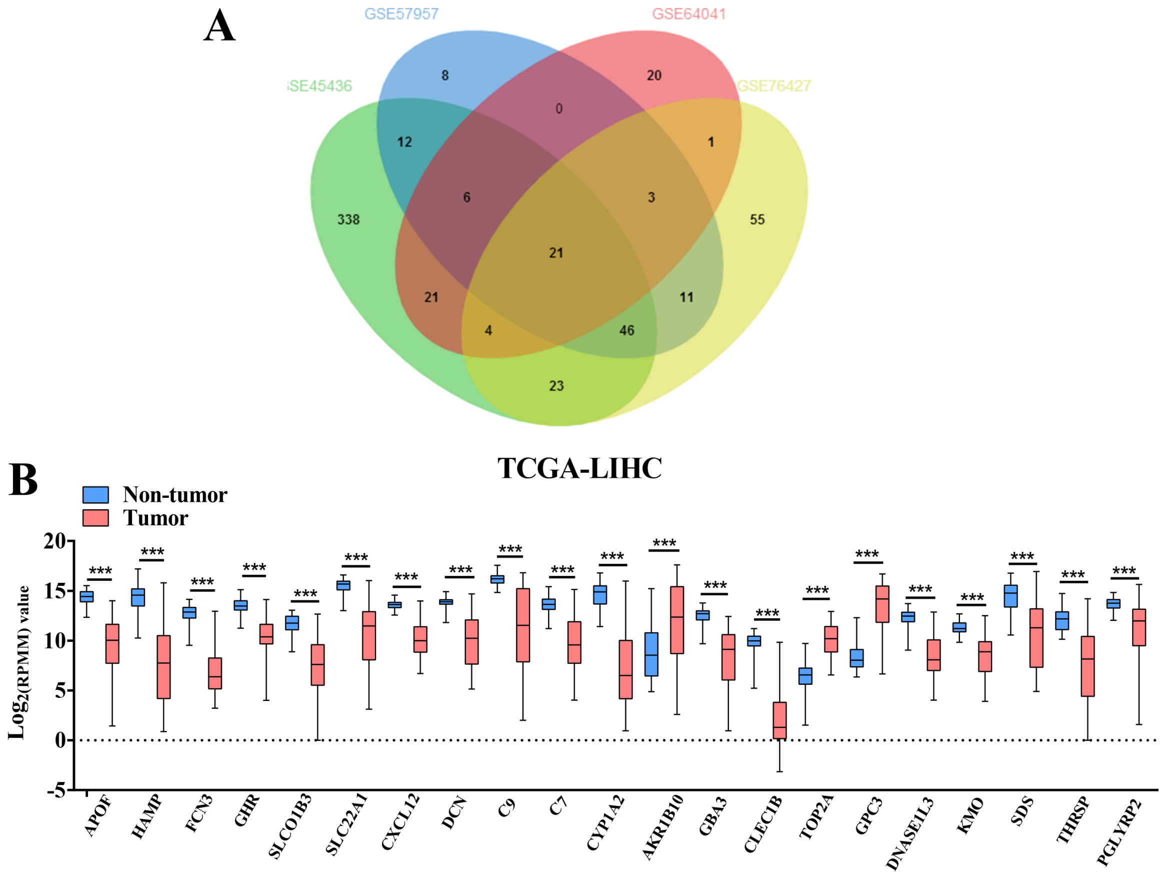

Supplementary Fig. 2

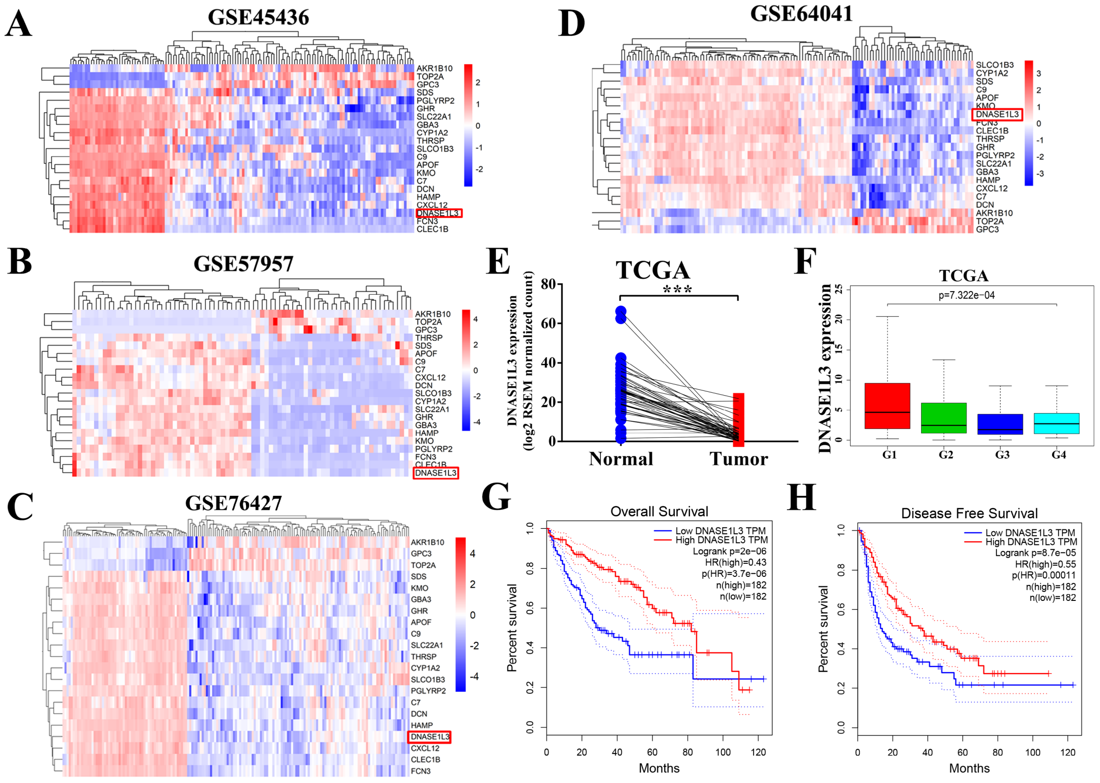

Supplementary Fig. 3

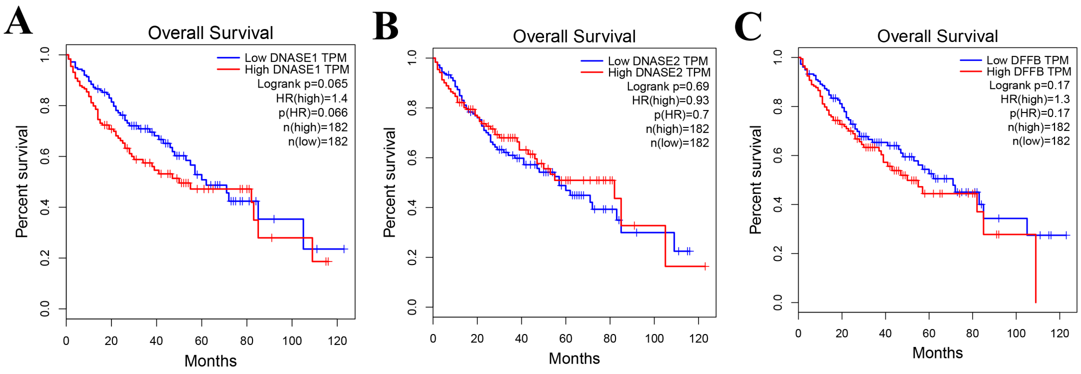

Supplementary Fig. 4

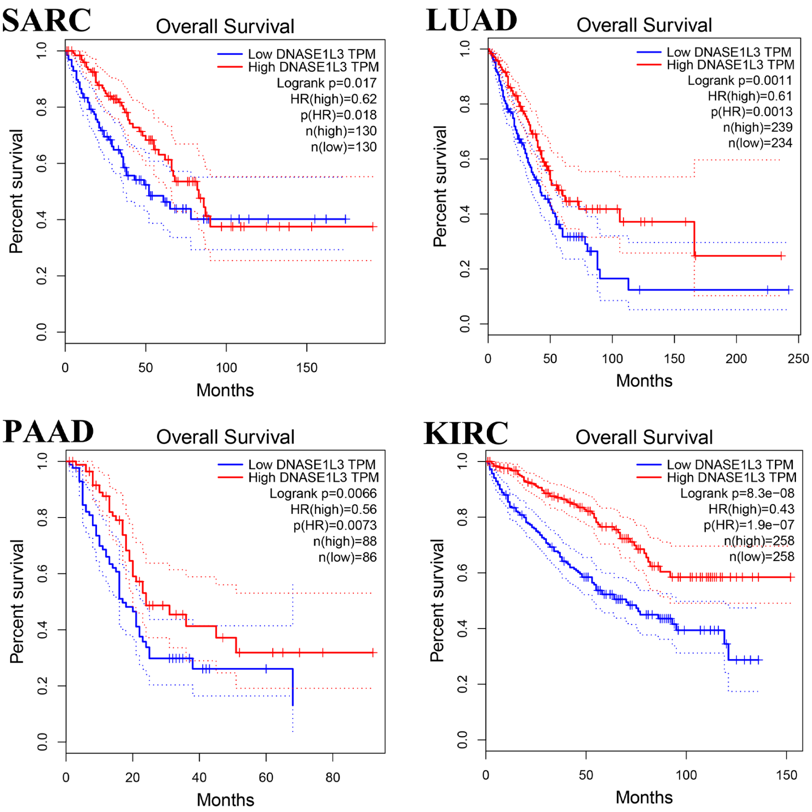

Supplementary Fig. 5

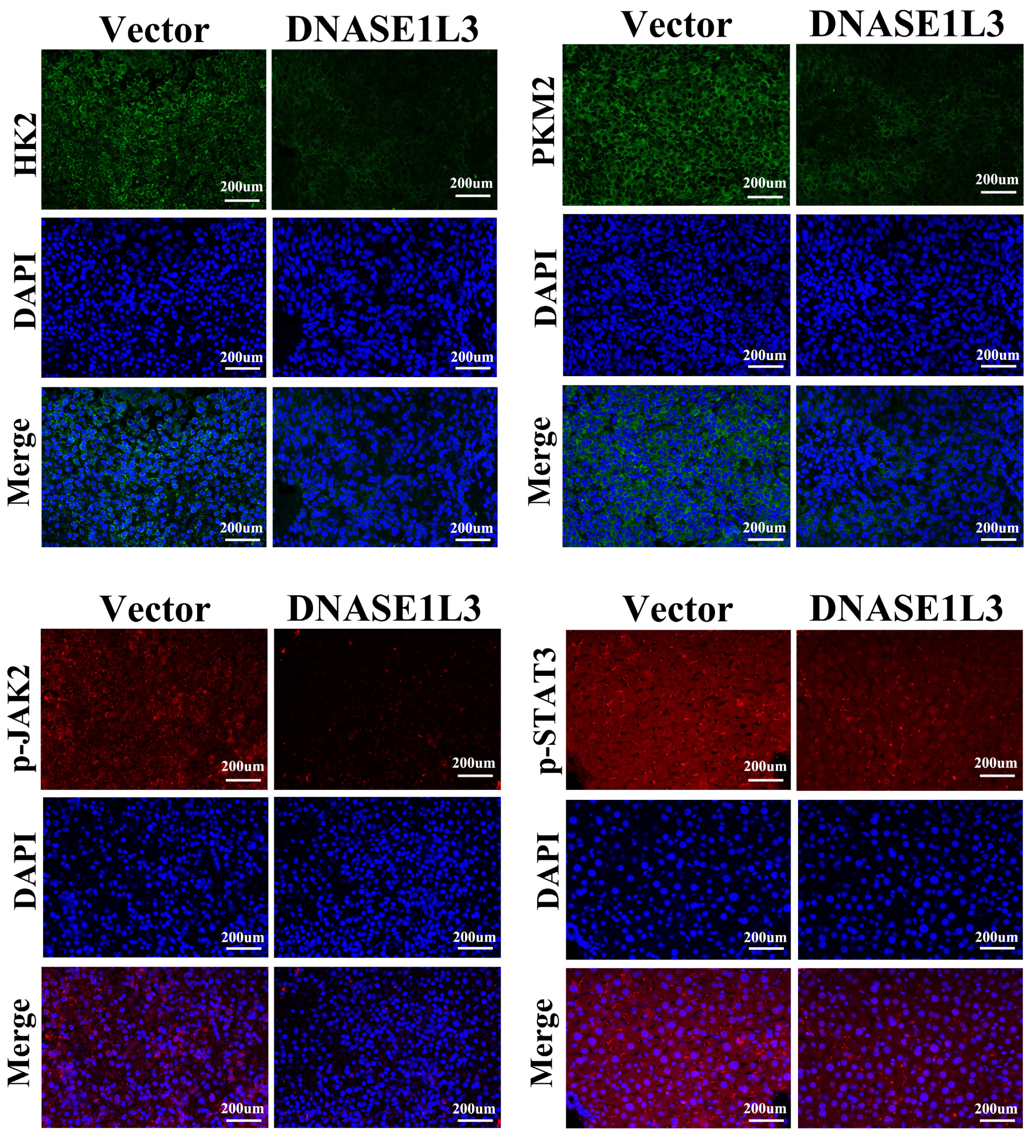

Supplementary Fig. 6

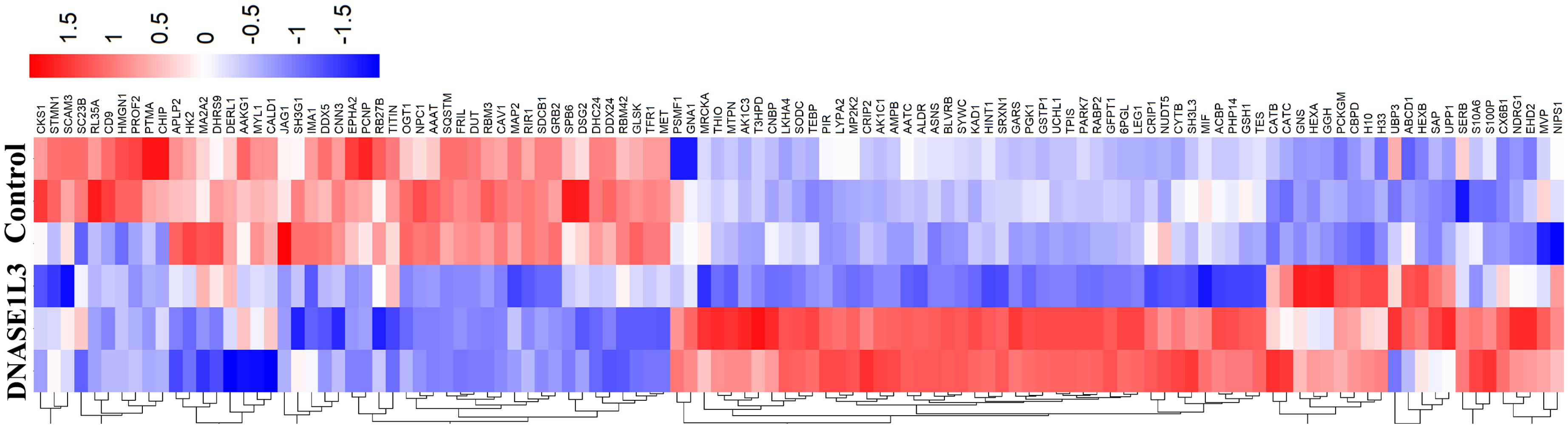

Supplementary Fig. 7

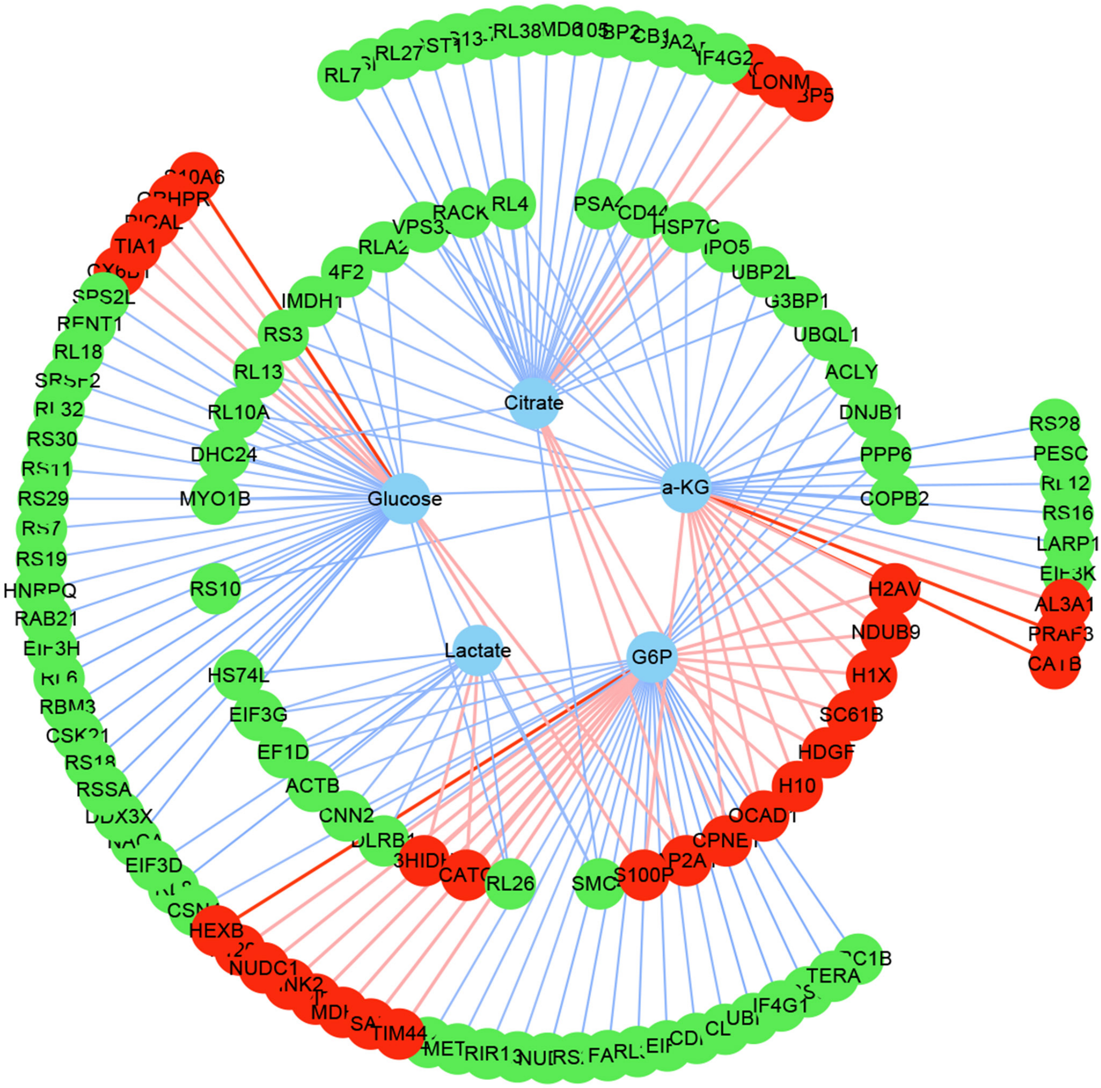

Supplementary Fig. 8

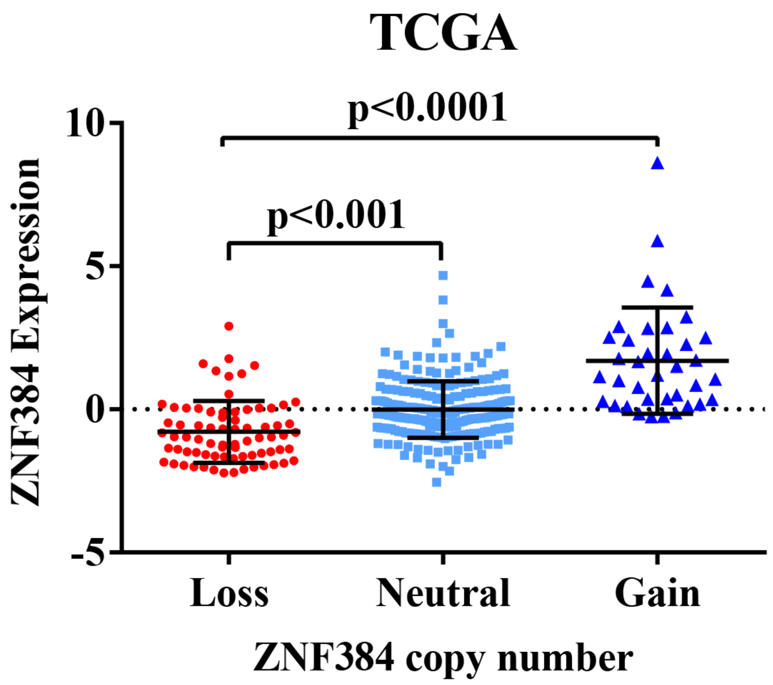

Supplementary Fig. 9

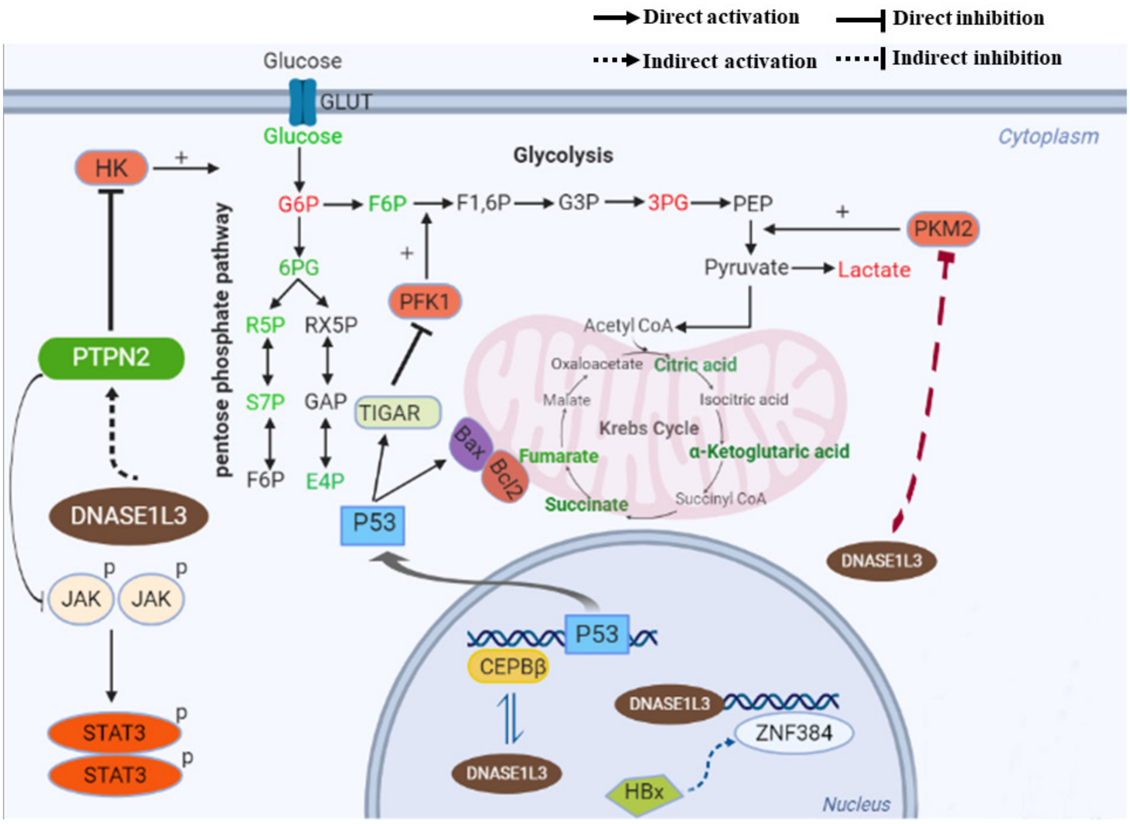

Supplementary Table 1. Primer sequences and target sequences

Target gene	5'-Sequence-3'
DNASE1L3	F: TCTCCTCCTCTCCATCCA
	R: CCTGTTGTTGCTGTCCTT
ZNF384	F: AAAGCACAGGGAAGCAGAAA
	R: TTGTCCATATCTTGCCATCTG
GAPDH	F: GTCTCCTCTGACTTCAACAGCG
	R: ACCACCCTGTTGCTGTAGCCAA
P53	F: CCTCCTCAGCATCTTATCC
	R: ACAAACACGCACCTCAAA
CEBPβ	F: TGAAGACGGATTGCCCTCATT
	R: GCTGGTGCCAGTAAGAGCTT
HK2	F: TCCCCTGCCACCAGACTA
	R: TGGACTTGAATCCCTTGGTC
PFK1	F: GCCATCAGCCTTTGACAGA
	R: CTCCAAAAGTGCCATCACTG
LDHB	F: AACTTGCTCTTGTGGATGT
	R: CTTCTTGCTGACGGACTC
PKM2	F: CGTCTGAACTTCTCTCATGGAA
	R: ATGGGGTCAGAAGCAAAGC
GLUT1	F: CTTTGTGGCCTTCTTTGAAGT
	R: CCACACAGTTGCTCCACAT
CS	F: GAAGTGCTTGTCTGGCTAA
	R: CCGTCCTGAGTTGAGTGT
TIGAR	F: TTCCTTACCAGCCACTCT
	R: AACTTCTCTTCCTTCCTCAA
PTPN2	F: CATCTCTGCCTTGTCTCTT
	R: GCTGGTCTTGAACTCCTG
HKDC1	F:TAAGGCACGAGGAGTTCA
	R: GTCATAGGCACAGGTCATC
HBx-F	TATGTCGACATGGCATCAATGCAGAAGCTGATC
	TCAGAGGAGGACCTGATGGCTGCTAGGCTGTGC
HBx(full-length)-R	TATCCGCGGGGCAGAGGGGAAAAAGTTGTTG
siZNF384	GCGUCCGUUACCCAGAAUATT
	UAUUCUGGGUAACGGACGCTT
siDNASE1L3#1	GGCTTGGAAGAAACACATA
siDNASE1L3#2	GCATAACGTACAACTATGT

Supplementary Table 2. Primary antibodies implied for Western blotting and IHC analysis

**Antibody**	**Company (Cat. No.)**	**Working concentrations**	
DNASE1L3	ThermoFisher (PA5-30006)	WB: 1/1000	IHC: 1/200
DNASE1L3	Proteintech (67041-1-Ig)	WB: 1/500	IHC: 1/100
Myc tag	Abcam(ab18185)		IP: 1/100
Bax	Abcam(ab32503)	WB: 1/1000	IHC: 1/200
Bcl2	Abcam(ab59348)	WB: 1/1000	
C-Myc	Abcam (ab32072)	WB: 1/1000	
Caspase3	Abcam (ab13847)	WB: 1/1000	
C-Caspase3	Abcam (ab2302)	WB: 1/1000	IHC: 1/200
CyclinD1	Abcam (ab40754)	WB: 1/2000	
HK2	Proteintech (22029-1-AP)	WB: 1/500	IP: 1/100
PKM2	Proteintech (15822-1-AP)	WB: 1/500	
LDHA	Proteintech (66287-1-Ig)	WB: 1/1000	
PDH	Proteintech (18068-1-AP)	WB: 1/500	
GLUT2	Proteintech (20436-1-AP)	WB: 1/500	
G6PD	Proteintech (66373-1-Ig)	WB: 1/500	
GAPDH	Proteintech (60004-1-Ig)	WB: 1/5000	
Ki67	Abcam (ab16667)		IHC: 1/200
PTPN2	Abcam (ab227916)	WB: 1/1000	IP: 1/100
P53	Proteintech (60283-2-Ig)		
CEBPβ	SANTA (sc-56637)	WB: 1/1000	IP:1/100
ZNF384	Abcam (ab176689)	WB: 1/2000	
JAK2	Abcam (ab108596)	WB: 1/5000	
p-JAK2	Abcam (ab32101)	WB: 1/2000	IF: 1/500.
STAT3	Abcam (ab119352)	WB: 1/2000	
p-STAT3	Abcam (ab76315)	WB: 1/2000	IF: 1/500
β-actin	Proteintech (66009-1-lg)	WB: 1/5000	

Supplementary Table 3. The relationship between DNASE1L3 and clinical features of HCC patients

Clinical features		DNASE1L3 expression		
	**Cases**	**Low**	**High**	**P-value**
	80	40	40	
**Gender**				
Male	54	26	28	0.633
Female	26	14	12	
**Age, years**				
≥median,65	43	21	22	0.823
< median, 65	37	19	18	
**Histologic grade**				
Well or moderate	48	18	30	**0.0062***
Poor	32	22	10	
**Edmondson-Steiner**				
I-II	60	25	35	**0.0098***
III-IV	20	15	5	
**BCLC Stage**				
A	65	29	36	**0.045***
B+C	15	11	4	
**Tumor size**				
≥5cm	33	22	11	**0.013***
< 5cm	47	18	29	
**Serum AFP(μg/L)**				
≥400	34	19	15	0.366
< 400	46	21	25	
**Tumor number**				
Sigular	63	28	35	0.056
Multifocal	17	12	5	
**Vascular invasion**				
Yes	13	10	3	**0.034***
No	67	30	37	

Supplementary Table 4. The correlation between DNASE1L3 expression and 13 potential transcriptional factors in TCGA-LIHC

**Gene Name**	**r**	Gene **N**ame	r
ZNF384	-0.455	ELF3	-0.14
CTCF	-0.04	**ETV5**	-0.16
EGR2	0.17	**KLF16**	-0.15
KLF9	0.26	**MEF2B**	-0.12
**MIXL1**	-0.06	MXI1	0.12
NRF1	-0.19	RBPJ	-0.09
RUNX3	0.13		
